# Characterisation of putative lactate synthetic pathways of *Coxiella burnetii*

**DOI:** 10.1371/journal.pone.0255925

**Published:** 2021-08-13

**Authors:** Janine Hofmann, Mebratu A. Bitew, Miku Kuba, David P. De Souza, Hayley J. Newton, Fiona M. Sansom

**Affiliations:** 1 Faculty of Veterinary and Agricultural Sciences, Asia-Pacific Centre for Animal Health, Melbourne Veterinary School, University of Melbourne, Parkville, Victoria, Australia; 2 Department of Pathology, Microbiology and Immunology, University of California, Davis, California, United States of America; 3 Department of Microbiology and Immunology, University of Melbourne at the Peter Doherty Institute for Infection and Immunity, Melbourne, Victoria, Australia; 4 Metabolomics Australia, The Bio21 Molecular Science and Biotechnology Institute, University of Melbourne, Parkville, Victoria, Australia; University of Arkansas for Medical Sciences, UNITED STATES

## Abstract

The zoonotic pathogen *Coxiella burnetii*, the causative agent of the human disease Q fever, is an ever-present danger to global public health. Investigating novel metabolic pathways necessary for *C*. *burnetii* to replicate within its unusual intracellular niche may identify new therapeutic targets. Recent studies employing stable isotope labelling established the ability of *C*. *burnetii* to synthesize lactate, despite the absence of an annotated synthetic pathway on its genome. A noncanonical lactate synthesis pathway could provide a novel anti-*Coxiella* target if it is essential for *C*. *burnetii* pathogenesis. In this study, two *C*. *burnetii* proteins, CBU1241 and CBU0823, were chosen for analysis based on their similarities to known lactate synthesizing enzymes. Recombinant GST-CBU1241, a putative malate dehydrogenase (MDH), did not produce measurable lactate in *in vitro* lactate dehydrogenase (LDH) activity assays and was confirmed to function as an MDH. Recombinant 6xHis-CBU0823, a putative NAD^+^-dependent malic enzyme, was shown to have both malic enzyme activity and MDH activity, however, did not produce measurable lactate in either LDH or malolactic enzyme activity assays *in vitro*. To examine potential lactate production by CBU0823 more directly, [^13^C]glucose labelling experiments compared label enrichment within metabolic pathways of a *cbu0823* transposon mutant and the parent strain. No difference in lactate production was observed, but the loss of CBU0823 significantly reduced ^13^C-incorporation into glycolytic and TCA cycle intermediates. This disruption to central carbon metabolism did not have any apparent impact on intracellular replication within THP-1 cells. This research provides new information about the mechanism of lactate biosynthesis within *C*. *burnetii*, demonstrating that CBU1241 is not multifunctional, at least *in vitro*, and that CBU0823 also does not synthesize lactate. Although critical for normal central carbon metabolism of *C*. *burnetii*, loss of CBU0823 did not significantly impair replication of the bacterium inside cells.

## Introduction

*Coxiella burnetii* is the Gram negative bacteria that causes the multifaceted human disease Q fever, an ever-present global public health threat [1]. Endemic and hyperendemic regions experience regular cases of Q fever. Sporadic outbreaks also transpire, the largest being in The Netherlands between 2007 and 2010, which emphasized both the serious health and economic impact of Q fever [2–4]. In particular, the more insidious chronic Q fever can cause significant morbidity and potentially death [5]. Current therapeutics can be problematic due to contra-indications or long courses so there is considerable need for novel drugs to treat Q fever effectively [1].

Recent stable isotope labelling studies demonstrated ^13^C-label incorporation into lactate by *C*. *burnetii*, despite the apparent lack of a known lactate synthesis pathway within the *C*. *burnetii* genome [6–8]. Lactate production can be beneficial to bacteria by several means. It can be the end product of efficient metabolism pathways, play a role in redox and energy homeostasis, be metabolized an alternative carbon source, or modify host immune response [9–18]. The ability to synthesize lactate has been linked to virulence in disparate species such as *Bacillus cereus*, *Staphylococcus aureus*, *Neisseria gonorrhoeae*, and lactic acid bacteria *Enterococcus faecalis*, *Streptococcus pyogenes*, *Streptococcus mutans* and *Streptococcus pneumoniae* [13–15,18–22]. Furthermore, inhibitors of a unique lactate synthesis enzyme of *Cryptosporidium parvum* reduced replication and pathogenicity [23]. As yet, the method and the essentiality of lactate synthesis to *C*. *burnetii* replication has not been investigated. Should unusual enzyme(s) responsible for lactate production in *C*. *burnetii* be determined and found to play an important role in the organism’s metabolism and pathogenicity, they may represent useful future anti-*Coxiella* targets.

**Fig 1:**
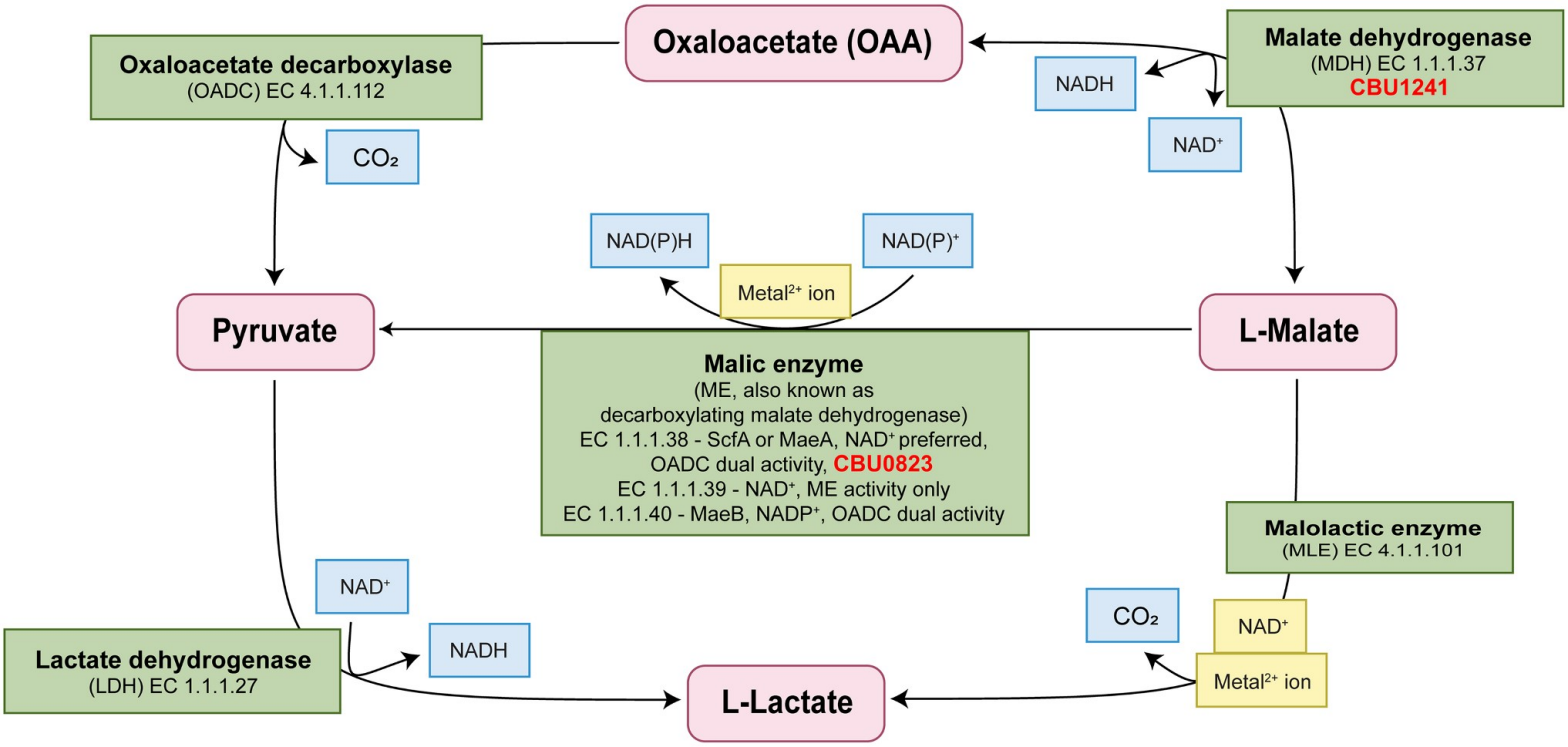
Schematic representation of enzymes commonly involved in 2-keto acid metabolism and their corresponding reactions. The enzymes, shown in green boxes, may require cofactors, shown in gold boxes. Additional substrates and products are shown in blue boxes. CBU0823 and CBU1241 have been included with their respective putative annotations. Adapted from [35].

Lactate dehydrogenases (LDHs) are widely distributed within prokaryotic and eukaryotic organisms, but no LDH is annotated on the *C*. *burnetii* genome. LDHs produce lactate from pyruvate and regenerate NADH from NAD^+^ in the process [9]. An LDH is also absent from genome annotations of the close *C*. *burnetii* relative *Legionella pneumophila* and some strains of fellow acidophile *Helicobacter pylori* [24,25]. LDHs share a common ancestor with malate dehydrogenase enzymes (MDHs) within the same superfamily of dehydrogenases (Fig 1) [26]. Their highly similar structures, containing many conserved residues, can result in substrate flexibility [27–29]. Some can use multiple substrates in their native state, others can be manipulated to switch substrate preferences with very few residue substitutions [26–32]. This feature is more widespread in LDHs than MDHs [27,33]. The *C*. *burnetii* gene *cbu1241*, annotated as a putative malate dehydrogenase, has previously been shortlisted as a potential virulence factor that is also expected to play a vital metabolic role and thus provides a promising anti-*Coxiella* drug target [34].

Similarly, malic enzymes (MEs) and malolactic enzymes (MLEs) are closely related enzymes capable of catalyzing multiple reactions (Fig 1) [35–37]. MEs act to decarboxylate malate to pyruvate, whereas MLEs decarboxylate malate to lactate without reduction of an essential NAD^+^ cofactor (Fig 1) [37,38]. MEs are broadly distributed throughout eukaryotes and prokaryotes, with the exception of most lactic acid bacteria, whereas malolactic enzymes (MLEs) are yet to be found outside lactic acid bacteria [39–41]. Substrate flexibility has been demonstrated for numerous members of both MEs and MLEs, with the MLE of *Oenococcus oeni* capable of particularly diverse catalytic activity, possessing MDH, LDH and ME functions in addition to MLE function [35,38,42–45].

In this study, we analyzed bioinformatic information regarding two *C*. *burnetii* genes *cbu1241*, annotated as a putative malate dehydrogenase, and *cbu0823*, annotated as a putative NAD^+^-dependent malic enzyme, in relation to likely substrate preferences. We then characterized the biosynthetic capabilities of recombinant CBU1241 and CBU0823 *in vitro* using both spectrophotometric absorbance measurements and gas chromatography-mass spectrometry (GC-MS) analysis, with a particular focus on the ability of these enzymes to synthesize lactate.

Furthermore, we investigated the involvement of *cbu0823* in lactate biosynthesis *in vivo* utilising stable isotope labelling techniques and a previously constructed *cbu0823* transposon mutant [46]. This approach also provided additional information as to the broader effect of a non-functional malic enzyme on the central carbon metabolism of *C*. *burnetii*. Finally, we examined the necessity of *cbu0823* for efficient replication of *C*. *burnetii* both axenically and intracellularly.

## Materials and methods

### Cell strains and culture conditions

*C*. *burnetii* Nine Mile RSA439 (phase II, clone 4) referred to as NMII RSA439 and strains derived from this parent were axenically cultured in liquid acidified citrate cysteine medium 2 (ACCM-2) [47] at 37°C in 2.5% O_2_ and 5% CO_2_. Antibiotic selection for *C*. *burnetii* transposon mutants was accomplished using chloramphenicol (3μg/ml) with additional kanamycin (350μg/ml) for complemented mutant strains. All plasmid construction was carried out in *Escherichia coli* DH5α and recombinant protein expression in *E*. *coli* JM109. *E*. *coli* strains were cultured in LB broth at 37°C with agitation unless otherwise specified, adding ampicillin (100 μg/ml), chloramphenicol (25μg/ml), or kanamycin (50 μg/ml) as required for plasmid selection. THP-1 human monocytic cells (ATCC TIB-202), were propagated in RPMI + GlutaMAX medium (Gibco, California, USA) supplemented with 5–10% fetal calf serum at 37°C in 5% CO_2_.

### Bioinformatics

Protein sequences were retrieved from the NCBI Protein Database within the Geneious Prime software. Sequence alignments were generated using Clustal Omega 1.2.2 [48] within Geneious Prime on default settings, with the alignment order set on “Group sequences by similarity”. Residues were compared to known key residues and motifs.

### Protein expression

Full-length *cbu0823* was amplified using PCR with gene-specific oligonucleotides (S1 Table) from purified *C*. *burnetii* genomic DNA. The gene was cloned into pQE-30 to produce recombinant CBU0823 with an N-terminus 6x-His tag. *cbu1241* was amplified with gene-specific oligonucleotides (S1 Table) and cloned into pGEX-4-T1 to produce an N-terminus glutathione S-transferase (GST)-fusion protein. A commercial wine making strain of *Oenococcus oeni* was purchased for PCR template as a freeze-dried preparation (Vitilac-F, Martin Vialatte, Magenta, France). The full-length malolactic enzyme was amplified using gene-specific oligonucleotides (S1 Table) then cloned into pQE-30. Cloned genes and affinity tags were confirmed with Sanger sequencing for all plasmids.

Proteins were expressed by culturing *E*. *coli* containing the relevant protein expression plasmid construct, the empty pQE-30 or pGEX-4-T1 plasmids (negative control) in 500 ml LB broth. At OD_600_ 0.6–0.8, protein expression was induced using 0.5 mM Isopropyl β-d-1-thiogalactopyranoside (IPTG) for 16 hours with agitation at 19°C. Whole cell lysate was then analyzed for protein expression using SDS-PAGE in TGX Stain-Free protein gels (BioRad, California, USA) under denaturing conditions and immunoblotting using primary mouse anti-His IgG (Invitrogen, California, USA) and secondary sheep anti-mouse IgG (GE Healthcare, Illinois, USA) for His-tagged proteins or with anti-GST-HRP conjugate (GE Lifesciences, Illinois, USA) for GST-tagged proteins.

The purification procedure used was dependent on affinity tag and protein characteristics. Pellets containing 6xHis-CBU0823 and empty pQE-30 were resuspended in 10 ml 50 mM NaH_2_PO_4_/300 mM NaCl/10 mM imidazole pH 8.0 lysis buffer containing 1 mg/ml lysozyme, then incubated on ice for 30 minutes. 1 mM phenylmethylsulfonyl fluoride (PMSF) was added prior to overnight incubation at 4°C. Cells were sonicated and 1% Triton X-100 added before centrifugation. The resultant supernatant was dialyzed against the same lysis buffer overnight at 4°C, before being filtered and loaded onto 1 ml NTA-Ni agarose beads (QIAGEN, Hilden, Germany). Column washing with 50 mM NaH_2_PO_4_/300 mM NaCl/20 mM imidazole pH 8.0 and elution with 50 mM NaH_2_PO_4_/300 mM NaCl/250 mM imidazole pH 8.0 was performed as per manufacturer’s recommendations, except elution volume was increased to 12 ml [49]. All elutions were pooled and buffer exchanged into phosphate-buffered saline (PBS), then concentrated to a final volume of 5 ml using Amicon Ultra-15 UltraCel-30K MWCO Centrifugal Filter (Millipore, Massachusetts, USA) before storage at 4°C.

6xHis-*O*. *oeni* MLE (denoted 6xHis-oMLE) purification was carried out as above except buffers were altered to accommodate preferences of a previously described 6xHis-oMLE [35]. oMLE lysis buffer contained 100 mM HEPES/100 mM KCl/10 mM pH 6.5, wash buffer 100 mM HEPES/100 mM KCl/20 mM imidazole pH 6.5, and elution buffer 100 mM HEPES/100 mM KCl/250 mM imidazole pH 5.9. Elutions were buffer exchanged into oMLE storage buffer containing 100 mM HEPES/0.1 m MnCl_2_ pH 6.0.

Bacterial pellets containing GST-1241 and GST alone were resuspended in a PBS buffer pH 7.4 and cell lysis performed as above. The resultant supernatants were syringe filtered prior to loading onto Glutathione Sepharose 4B beads (GE Healthcare, Illinois, USA). PBS pH 7.4 was used as column wash before eluting with 50 mM Tris/10 mM reduced glutathione (Sigma, Massachusetts, USA) pH 8.0. GST-1241 and GST were buffer exchanged into PBS pH 7.4 as above, with the exception of an Amicon Ultra-15 UltraCel-10K MWCO Centrifugal Filter (Millipore, Massachusetts, USA) for the smaller GST protein.

Purified protein was visualized on TGX Stain-Free gels and immunoblotted for His-tagged or GST-tagged proteins as above to confirm the presence of the correct protein tags on purified proteins. Purified protein was quantified using Qubit^™^ Protein Assay Kit with the Qubit^™^ 3.0 Fluorometer (Invitrogen, California, USA) as per manufacturer’s instructions.

### Spectrophotometric enzyme activity assays

NADP^+^ and NADPH were sourced from Roche (Basel, Switzerland) and all other reagents were purchased from Sigma (Massachusetts, USA). Reactions were prepared in triplicate in a single tube and the respective protein added immediately prior to dispensing single reactions of 200 μl into 96-well plates. This set of reactions was repeated three times in total for each measurement point, with the mean of each set used to represent the experiment in data analysis. Assays were repeated a minimum of 3 times. Immediately after dispensing, measurement of light absorbance at 340 nm, with software pathlength correction on, was performed on either Synergy H1 Hybrid multi-mode reader (BioTek, Vermont, USA) or FLUOstar microplate reader (BMG Labtech, Ortenberg, Germany). Measurements were taken every minute for 10 measurements.

Within this paper, the standard MDH forward reaction was 2 mM oxaloacetate (OAA) and 0.5 mM NADH in PBS pH 7.4. The standard LDH forward reaction was considered to be 2 mM pyruvate and 0.5 mM NADH in PBS pH 7.4. This can also detect ME reverse activity. The standard ME forward reaction for this paper contained 3 mM malate, 2.5 mM NAD^+^ and 1 mM MnCl_2_ in PBS pH 7.4. This can also detect MDH enzyme reverse activity. To assess the necessity of the metal cation cofactor for 6xHis-CBU0823 activity, selected reactions were repeated with or without 1 mM MnCl_2_. Suitability of other 2+ metal ions for 6xHis-CBU0823 cofactor function was tested in the standard ME reaction by replacing MnCl_2_ with 1 mM of CaCl_2_, CuCl_2_, MgCl_2_, ZnSO_4_, FeSO_4_, or NiSO_4_. To examine cofactor preference, NAD^+^ was replaced with NADP^+^ in the standard ME assay and NADH with NADPH in the standard MDH assay.

Standard reactions contained 2 μg GST-CBU1241, 2 μg GST (negative control), 20 μg 6xHis-CBU0823, 28.8 μg 6xHis-oMLE (equivalent to amount used in [32]), or equivalent volume as 6xHis-oMLE of pQE-30 negative control. Commercially prepared enzymes were used as positive controls, namely 0.013 units MDH (from pig heart mitochondria, Roche, Basel, Switzerland) and 0.1 units LDH (from beef heart, Sigma, Massachusetts, USA). To verify 6xHis-oMLE protein activity, oMLE assays were performed at 45°C with oMLE storage buffer replacing PBS and altered substrate concentrations to reproduce the previous characterisation of this enzyme [35].

The effect of pH on enzyme activity was determined relative to pH 7.4 by adjusting the pH of the PBS buffer before mixing of assay reactions through the range pH 4 to 11 for 1 μg GST-CBU1241 and pH 5 to 11 for 2 μg 6xHis-CBU0823 per well.

Enzyme kinetic properties for GST-CBU1241 were assessed using 0.1 μg GST-CBU1241 per well. Kinetics for OAA were assessed by increasing OAA concentration from 0.1 mM to 5 mM while maintaining NADH at 0.5 mM. Kinetics for NADH were assessed by increasing NADH concentration from 0.05 mM to 1.5 mM while maintaining OAA at 0.6 mM.

Enzyme kinetic properties for 6xHis-CBU0823 were assessed using 2 μg 6xHis-CBU0823 per well. Kinetics for malate were assessed by increasing malate concentration from 0.5 mM to 10 mM in reactions while maintaining NAD^+^ at 2.5 mM and MnCl_2_ at 1 mM. Kinetics for NAD^+^ were assessed by increasing NAD^+^ concentration from 0.1 mM to 5 mM while maintaining malate at 3 mM and MnCl_2_ at 1 mM. Kinetics for MnCl_2_ were assessed by increasing MnCl_2_ concentration from 0.01 mM to 10 mM while maintaining malate at 3 mM and NAD^+^ at 2.5 mM.

Michaelis constant (K_m_) and maximal reaction velocity within the system (V_max_) were calculated for GST-CBU1241 for OAA and 6xHis-CBU0823 for malate, NAD^+^ and MnCl_2_ using substrate concentrations below inhibitory levels on a Lineweaver-Burke plot using nonlinear regression (straight line) within GraphPad Prism 9.0 on default settings. This equates to above 1.5 mM^-1^ OAA for GST-CBU1241, and above 0.3 mM^-1^ malate, above 1.25 mM^-1^ NADH and above 1 mM^-1^ MnCl_2_ for 6xHis-CBU0823. Rate constant (k_cat_) was calculated from V_max_. Substrate inhibitor constant (K_Si_) was calculated using equations from [50]. Briefly, using the calculated V_max_, K_Si_ was represented by the slope when v/(V_max_−v) was plotted against 1/[S] (reciprocal of the quotient velocity plot). For GST-CBU1241, this slope was calculated for 1/[OAA] between 0.5 and 4 mM^-1^. For 6xHis-CBU0823, the slope was calculated for 1/[malate] between 0 and 0.4 mM^-1^, for 1/[NADH] between 0 and 1.25 mM^-1^, and for 1/[MnCl_2_] between 0 and 1 mM^-1^.

### Enzyme activity assay with GC-MS product detection

200 μl reactions containing 3 mM malate, 5 mM NAD^+^ and 1 mM MnCl_2_ and either 54 μg 6xHis-CBU0823, 28.8 μg 6xHis-oMLE, 3 μl of negative control purification (equivalent volume as 6xHis-CBU0823) or 3 μl PBS of blank control were prepared, with six replicates of each. After 20 minutes, the reaction was stopped by adding 100 μl of the reaction to 300 μl 100% methanol, then centrifugation used to remove the protein. Internal standards of 1 nmol ^13^C_6_-sorbitol and 10 nmol ^13^C_5_,^15^N-labelled valine were added to a 40 μl aliquot of the previous supernatant before storage at -80°C. For GC-MS analysis, 5 μl of this aliquot was dried completely in glass vial inserts within a rotational vacuum concentrator (Concentrator *Plus*, Eppendorf, Hamburg, Germany), including a final drying step of 30 μl 100% methanol.

The samples were derivatized with methoxyamine (Sigma, Massachusetts, USA) and N-bistrimethylsilyltrifluoroacetamide (BSTFA) containing 1% trimethylchlorosilane (TMCS; Thermo Scientific, Massachusetts, USA) before metabolites were analyzed on the Agilent 6545 series quadrupole mass spectrometer (Agilent Technologies, California, USA), using a protocol described previously [51]. Metabolite identification was performed by comparing molecular mass and retention time to authentic standards using MassHunter software (Agilent Technologies, California, USA). Results for each replicate were normalized to the valine internal standard value.

### *C*. *burnetii* transposon mutant complementation

*C*. *burnetii cbu0823* transposon (Tn) mutant from a previously generated transposon mutant library [46] was clonally isolated on semi-solid ACCM-2 agarose containing chloramphenicol [52]. PCR screening using transposon- and gene-specific oligonucleotide pairings (S1 Table) confirmed the transposon insertion within the *0823*::Tn mutant 832 basepairs downstream of the start codon, previously documented in [52].

To create the plasmid for genetic complementation of the *0823*::Tn mutant, full length *cbu0823* was amplified using gene-specific oligonucleotides (S1 Table), then cloned into the *C*. *burnetii* complementation vector pJB-kan:3xFLAG-MCS. Sequence and FLAG tag was confirmed by Sanger sequencing.

The complemented *0823*::Tn mutant strain was generated by introducing the pJB-kan:3xFLAG-*cbu0823* plasmid into the *0823*::Tn mutant as described previously [53]. Recovered bacteria were plated onto ACCM-2 agarose with chloramphenicol and kanamycin [47]. After 7 days of incubation, colonies were picked into 1 ml ACCM-2 with chloramphenicol and kanamycin selection in 24-well plates. Wells containing visible growth were harvested and whole cell lysate screened for 3xFLAG-CBU0823 expression by immunoblotting with primary anti-FLAG antibody (Sigma, Massachusetts, USA) and secondary anti-mouse IgG (GE Healthcare, Illinois, USA).

### Stable isotope labelling analysis

The method used in Hauslein et al. [7] was chosen to investigate lactate biosynthesis in *C*. *burnetii* as it produced the highest stable isotope label incorporation into lactate of published works [6,7]. Five replicates of 20 ml ACCM-2 containing 5 mM ^13^C-U-glucose (Sigma, Massachusetts, USA), each inoculated with 2 x 10^6^ GE/ml of the relevant *C*. *burnetii* strain, were incubated for 7 days as previously described [7],then processed for metabolite extraction in methanol:water:chloroform 3:1:1 v/v as previously described [6].

Preparation of glass inserts, derivatization and GC-MS analysis was performed as for the enzyme activity GC-MS assay, except the whole aqueous phase sample was dried down. DExSI software [54] was used for metabolite identification by comparison with an in-house Metabolomics Australia library of authentic standards for molecular masses and retention times. The peak integrations for all relevant mass isotopologues were combined for every detected metabolite and corrected to natural background isotopic abundance to give fractional labelling. The fractional labelling was then normalized to the initial ^13^C-glucose level within each replicate. Isotopologues were graphed using DExSI applying the inbuilt natural isotopic abundance correction for 0% unlabelled biomass. Detected metabolites were mapped to known *C*. *burnetii* metabolic pathways [55].

### Intracellular replication assays in THP-1 cells

THP-1 cells were seeded at 5 x 10^5^ cells per well into 24-well plates, with or without sterile glass coverslips, differentiated into macrophage-like cells by treating with 10 nM phorbol 12-myristate 13-acetate (PMA) and incubated for 3 days. 7-day cultures of *C*. *burnetii* strains were pelleted and resuspended in PBS. Genome equivalents were quantified using a quantitative qPCR that targets *ompA* as described previously, using oligonucleotides listed in S1 Table [56]. Each well was infected with a multiplicity of infection (MOI) of 25 with *C*. *burnetii* resuspended in 500 μl RPMI + 5% FCS for 4 hours at 37°C 5% CO_2_. Each strain was prepared in triplicate per replicate and six independent biological replicates were performed.

After infection, all wells were washed with PBS, then, except for day 0, 500 μl RPMI + 5% FCS was added per well for incubation at 37°C 5% CO_2_. Bacteria were harvested on days 0, 1, 3, 5, and 7. With the exception of day 0, the media from each well was collected and pelleted for 15 minutes at 13 200 x g. Attached cells were lyzed with nuclease-free water for 20 minutes. The lyzed cells were scraped from the well and added to the pellet from the media, before repeat centrifugation. The resulting pellet was resuspended in 100 μl nuclease-free water and *C*. *burnetii* genome equivalents (GE) determined by the *ompA* qPCR. Fold change was calculated for each time point relative to day 0.

Immunofluorescence microscopy slides were prepared on day 3 post-infection. The media was removed from wells containing coverslips and cells fixed in 4% paraformaldehyde before being permeabilized with 0.05% saponin + 2% BSA in PBS for 1 hour, then washed three times with PBS. Primary antibodies against *C*. *burnetii* (Roy Laboratory, Yale University, Connecticut, USA) and LAMP-1 (Developmental Studies Hybridoma Bank, Iowa, USA) at 1:10,000 and 1:500 respectively were applied for one hour, before further washing with PBS. Secondary antibodies anti-mouse AlexaFluor 488 and anti-rabbit AlexaFluor 568 (Thermo Fisher Scientific, Massachusetts, USA) were, both at 1:3,000, were applied for one hour and the first PBS wash that followed contained 1:10,000 4′,6-diamidino-2-phenylindole (DAPI) (Invitrogen, California, USA). Dako Fluorescent Mounting Media (Agilent Technologies, California, USA) was used to mount coverslips to glass slides. Images were obtained using Leica 780 and 700 confocal microscopes (Biological Optical Microscopy Platform, University of Melbourne, Melbourne, Australia) and processed in ImageJ.

Vacuole size quantification was completed in ImageJ, by measuring vacuole area in μm^2^ as seen on the anti-LAMP channel of at least 50 vacuoles per replicate. Four of the six biological replicates had sufficient quality immunofluorescence images to allow for vacuole quantification.

### Statistical analysis

All graphs were prepared using Prism 9.0 (GraphPad, California, USA). Unpaired two-tailed *t*-tests were performed in Prism 9.0 to compare the means of each enzyme activity in the GC-MS detection activity assay, for each metabolite in the stable isotope labelling experiment, as well as average vacuole area. Ordinary one-way ANOVA was performed in conjunction with Tukey’s multiple comparisons test in Prism 9.0 to examine differences in cofactor preference of 6xHis-CBU0823. Statistical differences in the intracellular replication assays between strains at each time point were calculated in Excel using unpaired two-tailed Student’s *t*-tests. A threshold significance of *p <* 0.05 was used in all analyses.

## Results

### The CBU1241 amino acid sequence is consistent with other malate dehydrogenases and CBU1241 possesses malate dehydrogenase function *in vitro*

Clustal Omega alignment of select MDH and LDH protein sequences revealed that CBU1241 and the putative MDH from *Legionella pneumophila* str. Philadelphia 1 shared the greatest identity at 63%. CBU1241 was more similar to the putative *Thermus thermophilus* MDH (56% identity) and the pig cytoplasmic MDH isozyme (46% identity) than to *E*. *coli* MDH (24% identity) (S1A Fig and S2A Table). This subgrouping of *T*. *thermophilus* with eukaryotic cytoplasm MDHs, as well as *Mycobacterium* species, has been noted previously and *C*. *burnetii* and *L*. *pneumophila* MDHs appear to lie within the same subgroup [28].

**Fig 2:**
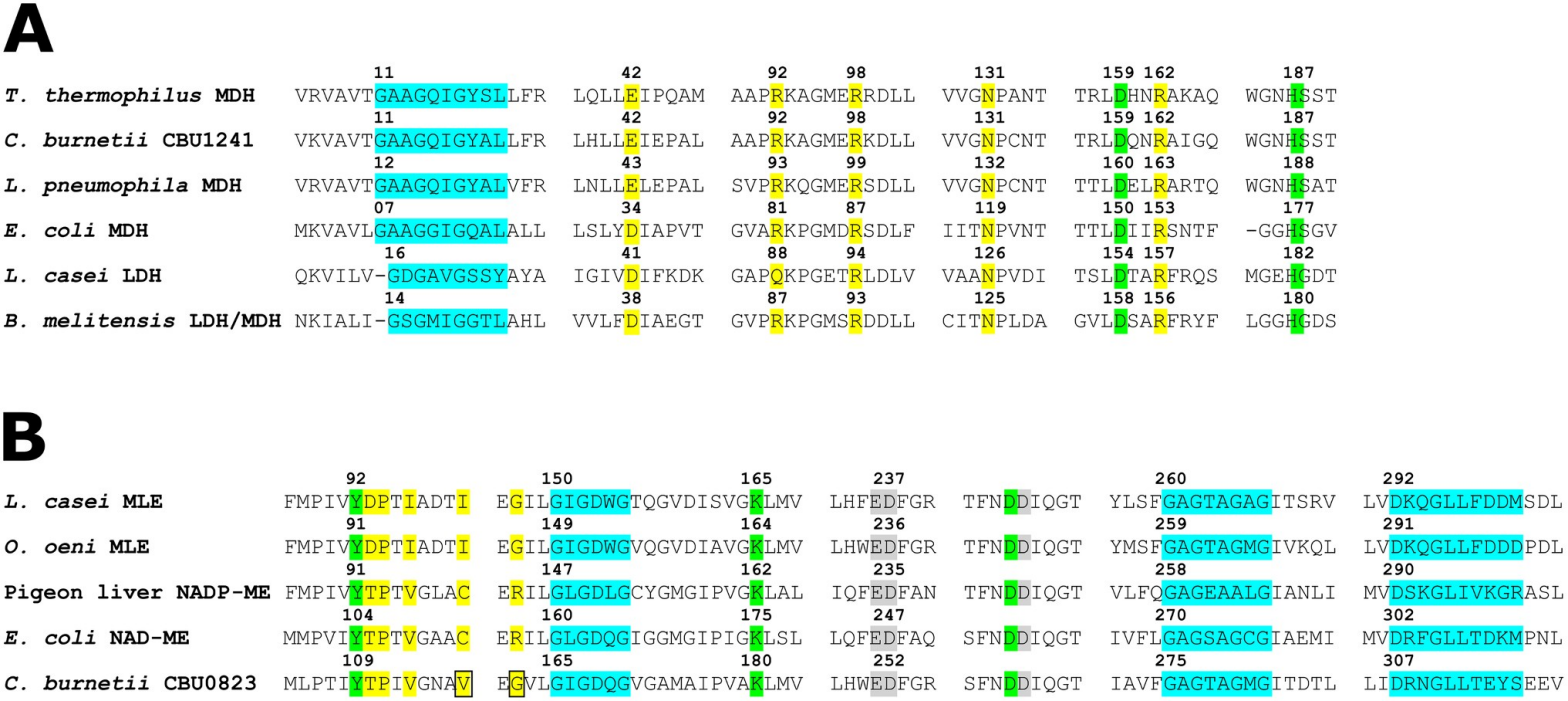
Key residues of CBU1241 and CBU0823. Protein sequence alignment sections containing key residues of (A) CBU1241 compared with MDHs and LDHs and (B) CBU0823 compared with MEs and MLEs. The NAD(P)-binding motifs (blue), substrate binding residues (yellow), catalytic activity residues (green), and metal binding residues (grey) are highlighted, and the boxes indicate unusual residues present within CBU0823 versus other bacterial MEs. Numbering refers to the residue position within the respective protein.

The presence of a Glu at position 42 and an Arg at position 92 (Fig 2) predicts that CBU1241 should have MDH function [28,33]. Other residues typical of the LDH and MDH superfamily involved in substrate binding (Arg98, Asn131 and Arg162) and proton transfer during catalytic action (Asp159 and His187) are conserved in CBU1241 [33,57–59]. No residues uncharacteristic of an MDH were identified in CBU1241.

**Fig 3:**
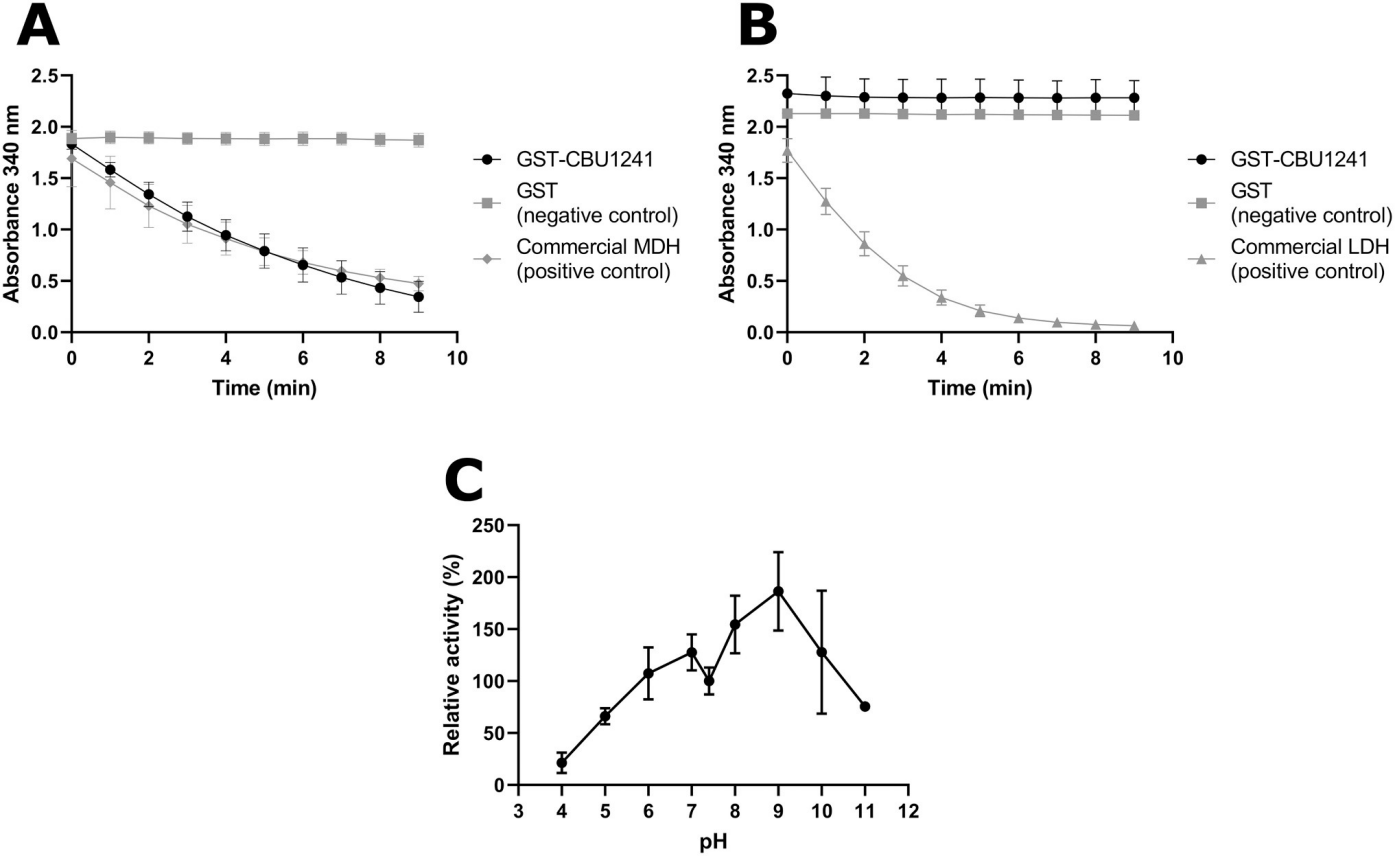
*In vitro* GST-CBU1241 activity assays for MDH, LDH or ME function. (A) MDH activity was exhibited by 2 μg GST-CBU1241 and 0.013 units commercial pig heart mitochondria MDH, whereas 2 μg GST alone exhibited no activity in the MDH standard assay containing 2 mM OAA and 0.5 mM NADH in PBS pH 7.4. (B) LDH activity was not observed for 2 μg GST-CBU1241 or 2 μg GST in the LDH standard assay containing 2 mM pyruvate and 0.5 mM NADH in PBS pH 7.4. Conversely, 0.1 unit of commercial LDH enzyme had measurable activity. (C) Enzyme activity relative to pH 7.4 over a range of pHs, measured using MDH standard assay containing 1 μg GST-CBU1241. Activity was measured using increase or decrease of light absorbance at 340 nm, correlating to NADH oxidation and NAD^+^ reduction respectively during enzyme activity. Error bars represent standard deviation from the mean from at least three independent experiments.

To assess the *in vitro* catalytic activities of CBU1241 in enzyme activity assays, recombinant GST-CBU1241 was expressed and purified (S2A Fig). When GST-CBU1241 was used in the MDH assay containing OAA and NADH, oxidation of NADH was observed as decreasing absorbance at 340 nm (Fig 3A). GST alone did not produce a measurable absorbance change (Fig 3A), suggesting that CBU1241 exhibited its predicted MDH function *in vitro*. The positive control, a commercially produced MDH, produced robust absorbance change in the same assay conditions (Fig 3A).

To evaluate lactate production *in vitro*, GST-CBU1241 was used in the LDH assay containing pyruvate and NADH. No change in absorbance was detected with either GST-CBU1241 or GST (Fig 3B). By contrast, the commercially produced LDH showed activity in standard assay conditions (Fig 3B). GST-CBU1241 MDH activity was maximal at pH 9.0, showing a preference for alkaline conditions (Fig 3D).

**Fig 4:**
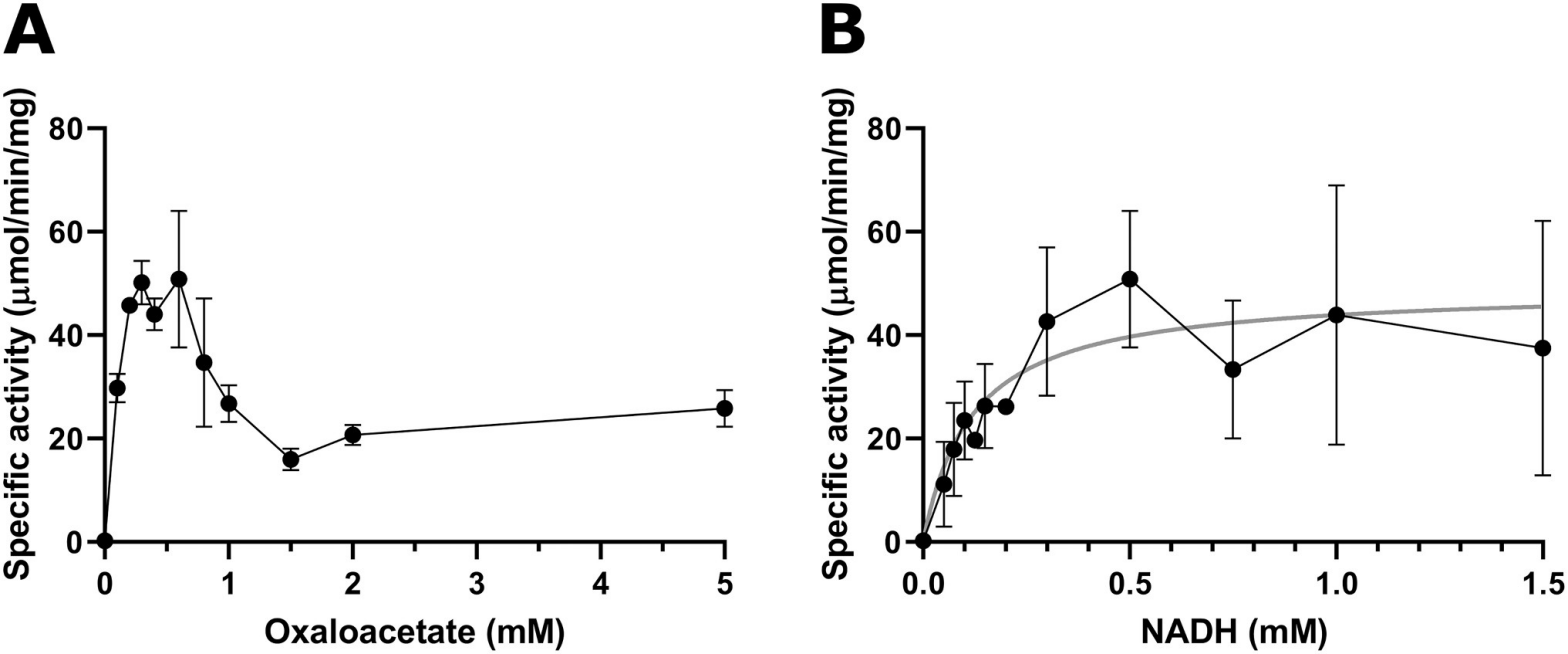
Michaelis-Menten plots of enzymatic activity for GST-CBU1241 in relation to substrates OAA (A) and NADH (B). Enzyme specific activity (μmol/mg per mg protein) measured for varying concentrations of OAA concentrations at 0.5 mM NADH (A), and varying NADH concentrations at 0.6 mM OAA (B). Error bars represent standard deviation from the mean from at least three independent experiments. Linear regression line of best fit for Michaelis-Menten kinetic equation is plotted in grey for NADH (B).

Enzyme kinetics showed that GST-CBU1241 activity was maximal between 0.3 and 0.6 mM OAA and substrate inhibition was observed above this concentration (Fig 4A). For OAA, K_m_ was calculated as 0.11 mM (95% CI 0.07–0.17), V_max_ as 63.61 μmol/min per mg (95% CI 54.61–76.22), k_cat_ as 65.20 s^-1^ (95% CI 55.98–78.13) and K_i_ was calculated as 1.25 mM (95% CI -0.85–3.39). Lineweaver-Burke and reciprocal of the quotient velocity plots are included in the supplementary information (S3A and S3B Fig). Enzyme activity more closely resembled classical Michaelis-Menten kinetics with increasing NADH concentrations (Fig 4B). The calculated K_m_ for NADH was 0.12 mM (95% CI 0.06–0.22), V_max_ 49.13 μmol/min per mg (95% CI 40.68–59.45), and k_cat_ 50.36 s^-1^ (95% CI 41.70–60.94). These values fall amongst those reported for other bacterial MDH proteins [60,61].

Together, this *in vitro* assessment demonstrated that GST-CBU1241 acts as an MDH, having no LDH activity, with a preference for alkaline conditions.

### CBU0823 shares many key residues with other bacterial malic enzymes and functioned as a malic enzyme and malate dehydrogenase *in vitro*

Clustal Omega alignment of ME and MLE protein sequences showed CBU0823 was most similar to one of the putative NAD^+^-dependent MEs of *L*. *pneumophila* Q5ZRB1 with 55% identity (S2B Table), which was expected given their close phylogeny [62]. CBU0823 also showed high identity with MLEs, 42% with the multifunctional *O*. *oeni* MLE and 41% with the MLE from *Lactobacillus casei* (S2B Table), permitting that CBU0823 could possess MLE function.

CBU0823 contained some key residue motifs not predicted by its NAD^+^-dependent ME annotation (Fig 2B). The presence of Gly162 in CBU0823 is more consistent with MLEs, as bacterial MEs usually contain an Arg, however, yeast MEs also contain a Gly at the equivalent residue [36]. Moreover, Val117 in the malate binding site of CBU0823 differed from both MLEs (Ile) and bacterial MEs (Cys), although all residues are hydrophobic [36,63].

In CBU0823, the GXGXXG motif beginning at Gly307 is consistent with other prokaryotic ME sequences and corresponds to the observed NAD^+^ binding site in resolved ME crystal structures [37,64]. Additional evidence for an NAD^+^-preference by CBU0823 can be found where the strictly conserved Asp341 and Gly344 is not surrounded by the motif of NADP^+^-preferring enzymes (Ser342, Lys343 and Arg351) [37,65,66].

Residues involved in catalytic activity (Tyr109, Lys180 and Asp274) and residues known to bind the metal cation cofactor (Glu251, Asp252, Asp275) were strictly conserved in CBU0823, reflecting their crucial functions [37,67,68].

**Fig 5:**
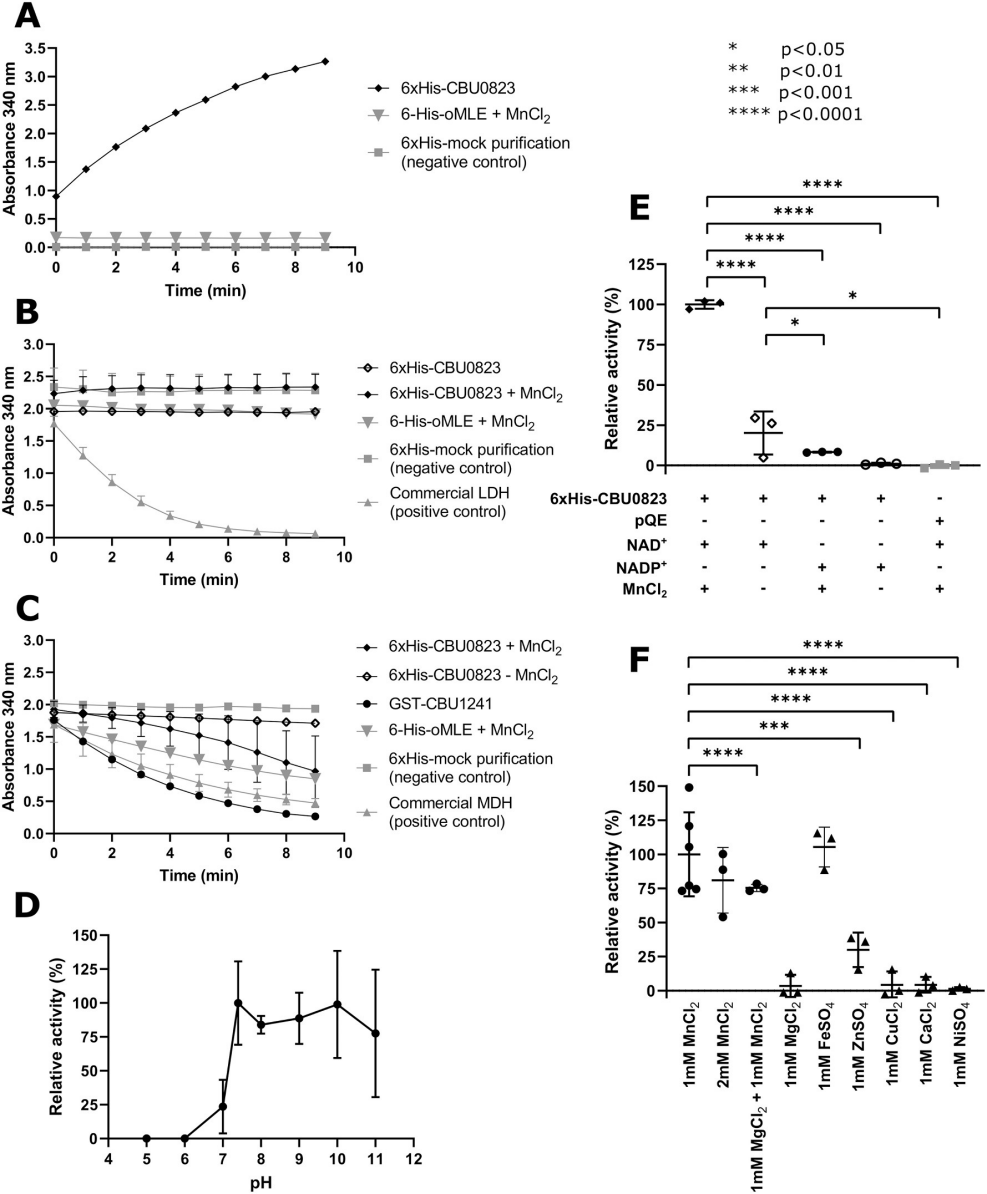
*In vitro* 6xHis-CBU0823 activity assays for ME (A), LDH (B) and MDH (C) function, including the influence of varied pH (D), and substituted cofactors (E and F). (A) ME activity was observed for 20 μg 6xHis-CBU0823 in the ME standard assay containing 3 mM malate, 5 mM NAD^+^, 1 mM MnCl_2_. 28.8 μg 6xHis-oMLE and 6xHis-mock purification negative control had no activity. (B) LDH activity was not detected for 20 μg 6xHis-CBU0823 in the LDH standard assay containing 2 mM pyruvate and 0.5 mM NADH, with or without the addition of MnCl_2_. 28.8 μg 6xHis-oMLE and 6xHis-mock purification also failed to show activity, while 0.1 units commercial LDH enzyme had measurable performance in standard conditions. (C) MDH activity was measured for 20 μg 6xHis-CBU0823 in the MDH standard assay containing 2 mM OAA, 0.5 mM NADH, with improved action on addition of 1 mM MnCl_2_. 0.013 units commercial pig heart mitochondrial MDH and 1 μg GST-CBU1241 had efficient activity in the assay and 6xHis-mock purification had no detectable activity. (D) Enzyme activity relative to pH 7.4 over a range of pHs, measured using ME standard assay containing 2 μg 6xHis-CBU0823. Activity was measured using increase or decrease of light absorbance at 340 nm, correlating to NADH oxidation and NAD^+^ reduction respectively during enzyme activity. (E) Removal of MnCl_2_ and/or substitution with NADP^+^ cofactor from the standard ME assay reduced 6xHis-CBU0823 activity. Conditions with significantly less activity are indicated. (F) Replacement of the MnCl_2_ cofactor with 1mM CaCl_2_, CuCl_2_, MgCl_2_, or ZnSO_4_ achieved varying levels of 6xHis-CBU0823 function. Those with significantly lower cofactor facility compared to 1 mM MnCl_2_ on ordinary one-way ANOVA are indicated (*p* < 0.05). Error bars represent standard deviation around the mean from at least three independent experiments.

To assess the *in vitro* catalytic activities of CBU0823, recombinant 6xHis-CBU0823 was expressed and purified (S2B Fig). When the 6xHis-CBU0823 recombinant protein was added to the ME assay containing malate, NAD^+^ and MnCl_2_, NAD^+^ reduction was observed as increasing absorbance at 340 nm, indicating 6xHis-CBU0823 exhibited the annotated ME function *in vitro* (Fig 5A). The pQE-30 negative control produced no absorbance change in this ME assay (Fig 5A).

Akin to GST-CBU1241, there was no change in absorbance when 6xHis-CBU0823 was used in the LDH standard assay, suggesting no lactate production (Fig 5B). Interestingly, 6xHis-CBU0823 was capable of oxidizing NADH in the presence of OAA in the standard MDH reaction (Fig 5C), indicating it possessed both ME and MDH activity.

6xHis-CBU0823 preferred increasingly alkaline conditions and activity was not detected in acidic conditions (Fig 5D). The ME activity of recombinant 6xHis-CBU0823 exhibited a strong preference for NAD^+^, as activity with NADP^+^ cofactor was significantly reduced to 8.23±0.24% to that with NAD^+^ (Fig 5E). This is consistent with other enzymes from the same enzyme class [69]. Removal of the Mn^2+^ cofactor also significantly reduced ME activity of 6xHis-CBU0823 to 20.14±13.38% (Fig 5E). Fe^2+^ effectively replaced Mn^2+^ as cofactor (Fig 5F). All other tested metals provided significantly reduced activity, with Zn^2+^ the next most proficient cofactor replacement with only 29.98±12.68% activity (Fig 5F).

**Fig 6:**
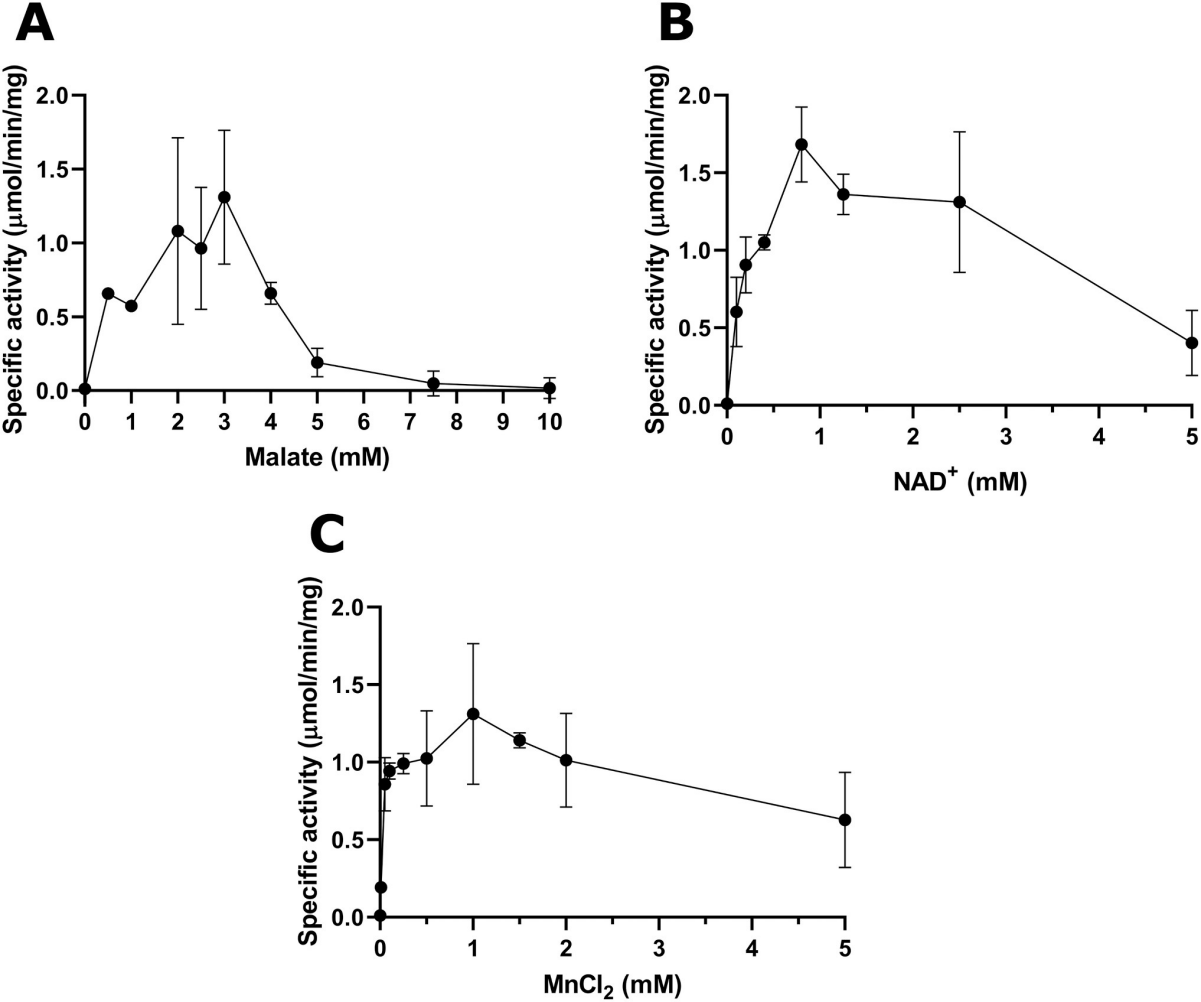
Michaelis-Menten plots of enzymatic activity for 6xHis-CBU0823 in relation to malate (A), NAD^+^ (B), and MnCl_2_ (C). Enzyme specific activity (μmol/min per mg protein) measured for (A) varying malate concentrations at 5 mM NAD^+^ and 1mM MnCl_2_, (B) varying NAD^+^ concentrations at 3 mM malate and 1 mM MnCl_2_, and (C) varying MnCl_2_ concentrations at 3 mM malate and 5 mM NAD^+^. Error bars represent standard deviation around the mean from at least three independent experiments.

Michaelis-Menten plots revealed substrate inhibition also occurred with 6xHis-CBU0823. Maximum activity was between 2 and 3 mM malate (Fig 6A), at 0.8 mM NADH (Fig 6B), and at 1 mM MnCl_2_ (Fig 6C). For malate, K_m_ was calculated as 0.40 mM (95% CI 0.01–1.25), V_max_ as 1.06 μmol/min per mg (95% CI 0.78–1.67), k_cat_ as 1.12 s^-1^ (95% CI 0.82–1.77) and K_i_ was calculated as 10.86 mM (95% CI -7.31–29.05). These values fall amongst those reported for other bacterial MDH proteins [60]. Lineweaver-Burk and reciprocal of the quotient velocity plots are included in the supplementary information (S3C and S3D Fig).

For NADH, K_m_ was calculated as 0.31 mM (95% CI 0.03-undefined), V_max_ as 2.06 μmol/min per mg (95% CI 0.78-undefined), k_cat_ as 2.18 s^-1^ (95% CI 0.83-undefined) and K_i_ was calculated as 6.30 mM (95% CI 0.27–12.33). Lineweaver-Burk and reciprocal of the quotient velocity plots are included in the supplementary information (S3E and S3F Fig).

For MnCl_2_, K_m_ was calculated as 0.06 mM (95% CI 0.04–0.09), V_max_ as 1.34 μmol/min per mg (95% CI 1.07–1.90), k_cat_ as 1.45 s^-1^ (95% CI 1.13–2.01) and K_i_ was calculated as 6.82 mM (95% CI -5.15–18.79). Lineweaver-Burk and reciprocal of the quotient velocity plots are included in the supplementary information (S3G and S3H Fig).

6xHis-oMLE did not perform well in the standard assay conditions (Fig 5A–5C). For example, in the ME standard reaction, 6xHis-oMLE did not have detectable activity. To confirm the purified protein could behave similarly to the previously published study, reactions were repeated with substrate concentrations as previously published at 45°C using oMLE storage buffer as diluent [35] (S4A–S4D Fig). 6xHis-oMLE activity was improved in these conditions. Notably, the observed reduction of OAA indicating MDH activity was not detected in the previous characterisation and represents yet more substrate flexibility of this versatile enzyme (S4B Fig) [35].

These *in vitro* data demonstrated that CBU0823 acts preferentially as an ME, converting malate to pyruvate. It is also capable of MDH activity but not of MLE or LDH activity. It has a strong preference for alkaline conditions and NAD^+^/NADH and Mn^2+^ as cofactors, though it will tolerate some substitutions.

### CBU0823 does not exhibit malolactic enzyme activity *in vitro*

**Fig 7:**
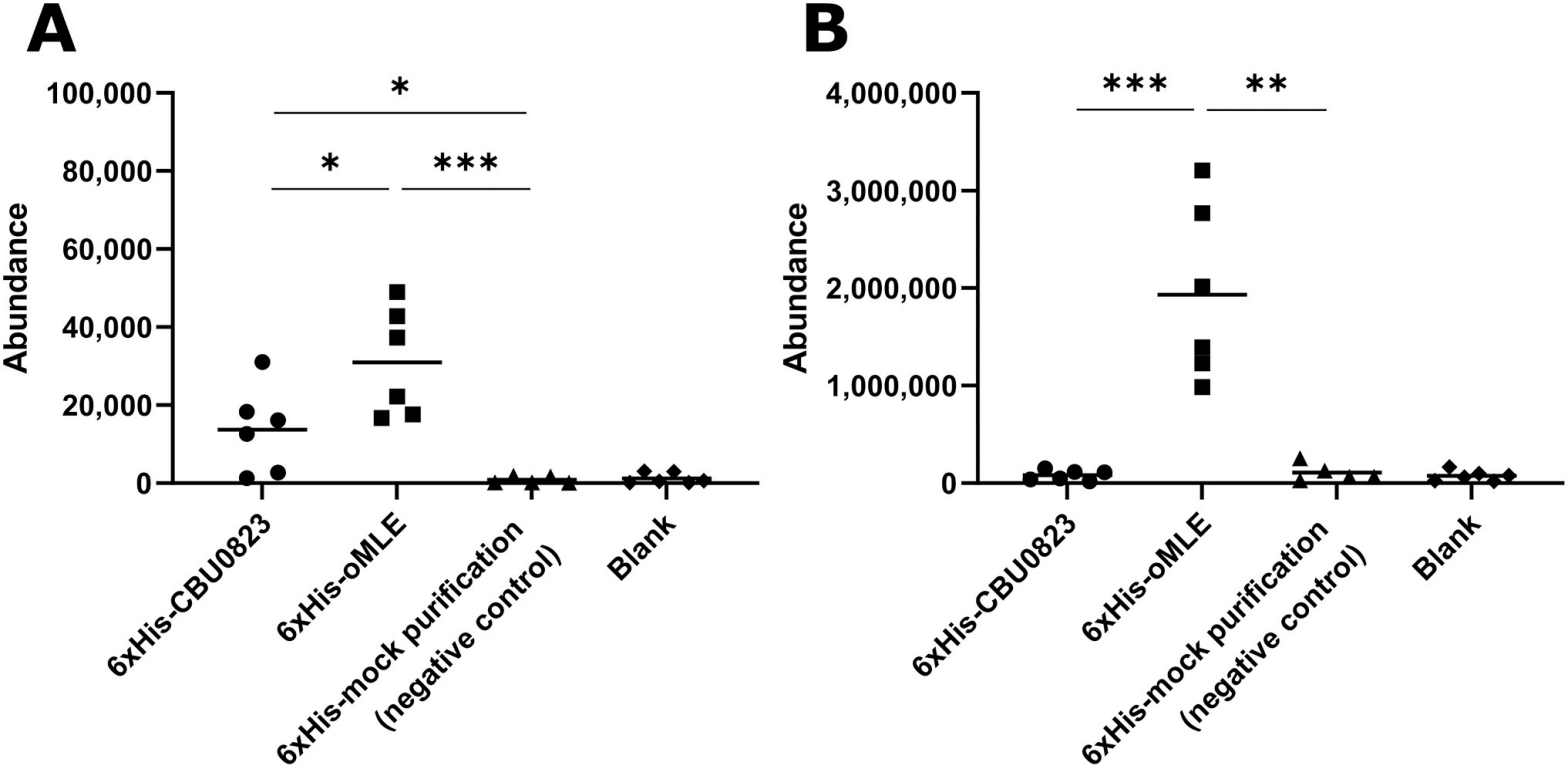
6xHis-CBU0823 failed to produce detectable lactate in *in vitro* assays. (A) Addition of 6xHis-oMLE produced significantly more pyruvate than 6xHis-CBU0823, and both produced significantly more than negative control and blank reactions. (B) Only 6xHis-oMLE yielded lactate amounts significantly above blank. Malic enzyme activity assays (3 mM malate, 5 mM NAD^+^ and 1 mM MnCl_2_) were started with either 54 μg 6xHis-CBU0823, 26 μg 6xHis-oMLE, or equivalent volume of mock purification (negative control) or PBS (blank). Reactions were stopped after 20 minutes. Each reaction was analyzed by GC-MS and mean values compared (* *p* < 0.05, ** *p* < 0.01, *** *p* < 0.001). Error bars represent standard deviation from the mean from six independent replicates.

The GC-MS analysis of *in vitro* activity indicated that 6xHis-oMLE could generate amounts of lactate and pyruvate significantly above baseline, corroborating the previously published work showing oMLE has ME and MLE activity (Fig 7) [35]. 6xHis-CBU0823 similarly produced pyruvate significantly above baseline (Fig 7A) supporting that malic enzyme activity was demonstrated in Fig 5A, but did not produce lactate above baseline levels (Fig 7B), suggesting it cannot act as an MLE. Comparatively 6xHis-CBU0823 generated significantly less pyruvate than 6xHis-oMLE, particularly as double the amount of 6xHis-CBU0823 enzyme was used. This suggests that despite the ME activity of CBU0823 being more efficient than its MDH activity, it is still less active than the malic enzyme activity of the multipurpose enzyme oMLE in these assay conditions. The instability of OAA precludes its detection by GC-MS, therefore we were unable to confirm whether CBU0823 could produce OAA by means of a reverse malate dehydrogenase activity.

### Loss of *cbu0823* causes no detectable change in ^13^C-incorporation into lactate

In order to evaluate the *in vivo* capacity of CBU0823 to synthesize lactate in *C*. *burnetii*, we wanted to compare stable isotope label enrichment in lactate in the *cbu0823* transposon insertion mutant to that of the wildtype strain. Following confirmation of the transposon insertion in the *0823*::Tn mutant, it was genetically complemented with pJB-kan:3xFLAG-*cbu0823* plasmid to provide constitutively expressed 3xFLAG-CBU0823. Expression of a correct sized product was confirmed on a western blot probed for FLAG tag (S2C Fig).

**Fig 8:**
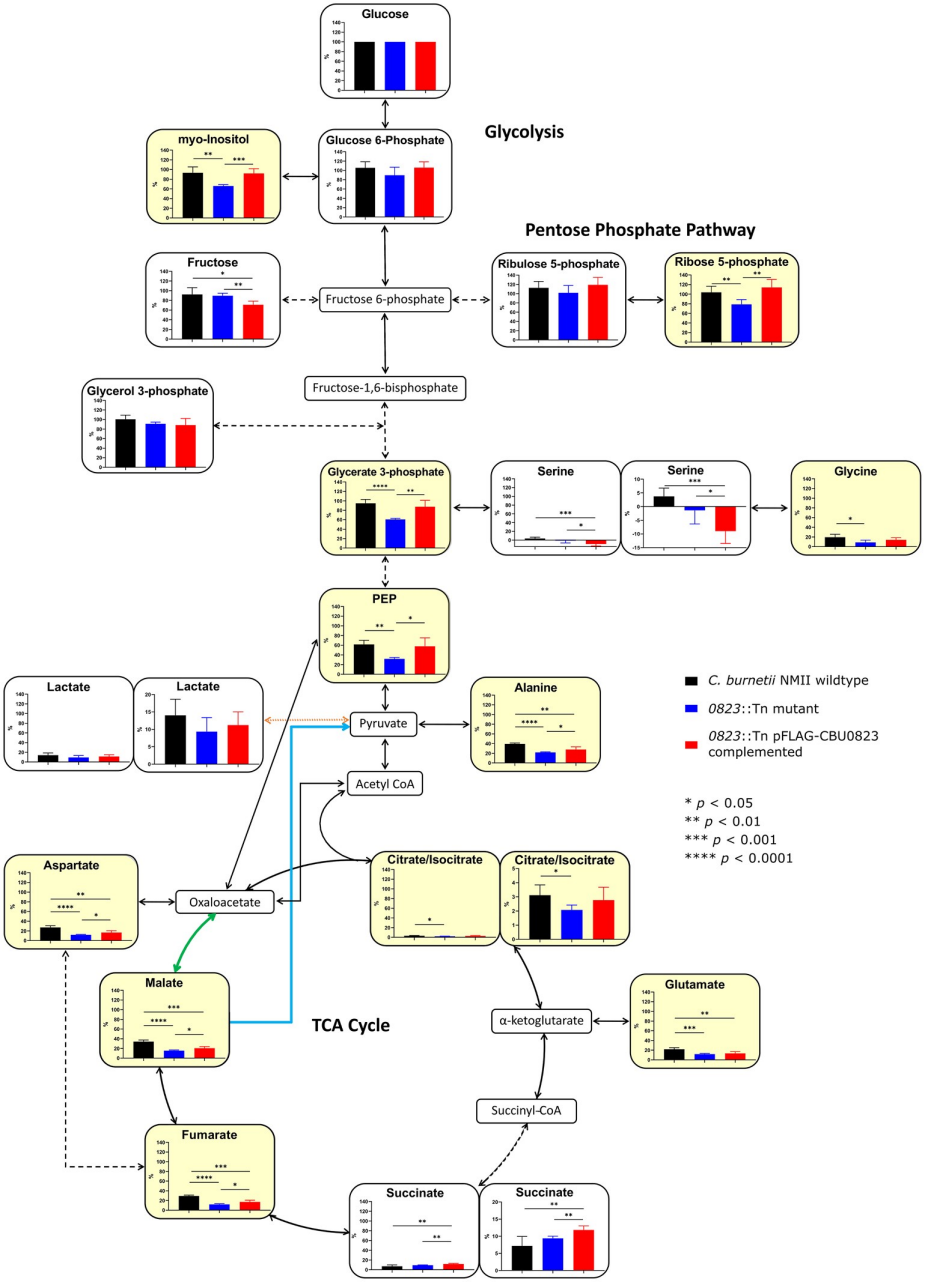
Loss of *cbu0823* caused alterations in carbon flux through glycolysis and the TCA cycle in *C*. *burnetii*. *C*. *burnetii* incubated with [^13^C]glucose supplemented ACCM-2 for 7 days before GC-MS analysis to determine ^13^C incorporated into the extracted metabolites. Mean label enrichment of each metabolite detected was compared between wildtype (black), *0823*::Tn mutant (blue), and *0823*::Tn pFLAG-CBU0823 complemented mutant (red) strains. Error bars represent standard deviation from five biological replicates. Metabolites with a significant difference between wildtype and the *0823*::Tn mutant are shaded in yellow. Solid lines represent a single enzyme and dotted lines represent multiple enzymes. The blue line denotes the activity of CBU0823 as a ME. The green line denotes CBU1241 as an MDH. The orange line represents an unestablished conversion of pyruvate to lactate by an LDH-type reaction. PEP = phosphoenolpyruvic acid.

The *C*. *burnetii* strains were cultured for 7 days in ACCM-2 containing [^13^C]glucose before harvest and analysis (Fig 8), conditions in which *C*. *burnetii* can incorporate labelling into lactate [7]. In wildtype, label enrichment into lactate was 14±4.6%, compared to 9.3±4.0% in the *0823*::Tn mutant and 11.2±3.8% in the *0823*::Tn pFLAG-CBU0823 complemented mutant. These ^13^C-incorporation levels were not statistically significantly different (*p* > 0.05), suggesting CBU0823 is not involved in lactate biosynthesis. The wildtype label enrichment in lactate detected in this study is similar to the previously published study, despite differing label enrichment calculation methodologies [7]. In the small amount of labelled lactate detected, all strains showed an isotopologue pattern of mostly M+2, with a small amount of M+1, once natural isotopic abundance correction was applied, suggesting the precursor might be a TCA cycle intermediate (S5 Fig). In contrast, Hauslein et al. observed a predominantly M+3, more suggestive of a pyruvate or glycolysis intermediate precursor [7].

### Loss of *cbu0823* alters central carbon metabolism of *C*. *burnetii*

The stable isotope labelling studies examining lactate biosynthesis advantageously provided information about the wider consequences of disabling *cbu0823* to *C*. *burnetii* central carbon metabolism. After 7 days incubation with [^13^C]glucose, the fraction labelled was significantly reduced in lower glycolysis intermediates within *0823*::Tn mutant compared to wildtype (Fig 8). For example, glycerate 3-phosphate within the *0823*::Tn mutant contained only 60.7±2.1% ^13^C-incorporation compared to 95.0±8.0% in wildtype and 87.6±13.5% in the *0823*::Tn pFLAG-CBU0823 complemented mutant. This suggests that overall the glycolytic pathway is less active in the *0823*::Tn mutant compared to wildtype, at least in its utilization of glucose.

The significant reduction in ^13^C-incorporation from [^13^C]glucose was also observed in TCA cycle intermediates in the absence of CBU0823. All metabolites contained significantly lower label enrichment in the *0823*::Tn mutant than in wildtype, except for succinate (Fig 8). For instance, label incorporation into malate, the substrate for CBU0823 and the product of CBU1241 in the *in vitro* assays, was 34.2±3.2% in wildtype yet only 15.4±1.3% in the *0823*::Tn mutant and 20.6±3.3% in the *0823*::Tn pFLAG-CBU0823 complemented mutant strain. Additionally, TCA cycle derivates aspartate and glutamate had lower label inclusion levels, suggesting the decreased fraction labelling continued into these derivative biosynthesis pathways as well. The M+2 isotopologue was most prevalent through detected TCA cycle intermediates (S5 Fig), supporting previous findings of carbon entering the TCA cycle via a fully labelled acetyl-CoA intermediate [7,70].

It is worth noting that the inconsistent pattern on label enrichment before and after citrate/isocitrate is likely due to citrate/isocitrate enrichment dilution by unlabelled citrate, present in large amounts in *C*. *burnetii* axenic media and remaining despite multiple washes, and is not related to any unusual TCA cycle direction within *C*. *burnetii*. The negative label enrichment in serine detected is physiologically impossible and probably detection of noise rather than true ^13^C-incorporation levels. The differences observed between wildtype and the *0823*::Tn pFLAG-CBU0823 complemented mutant strain are likely explained by the non-physiological, constitutive expression of pFLAG-CBU0823.

Taken as a whole, these data suggest the loss of CBU0823, a malic enzyme, within *C*. *burnetii* led to significant reductions in carbon flux from glucose through glycolysis and within the TCA cycle as well. This is potentially due to an inability to maintain adequate metabolic intermediates in the absence of the malic enzyme, particularly malate and acetyl coenzyme A (acetyl CoA), thus preventing metabolic processes from continuing as normal.

### *cbu0823* is not required for efficient *C*. *burnetii* replication

**Fig 9:**
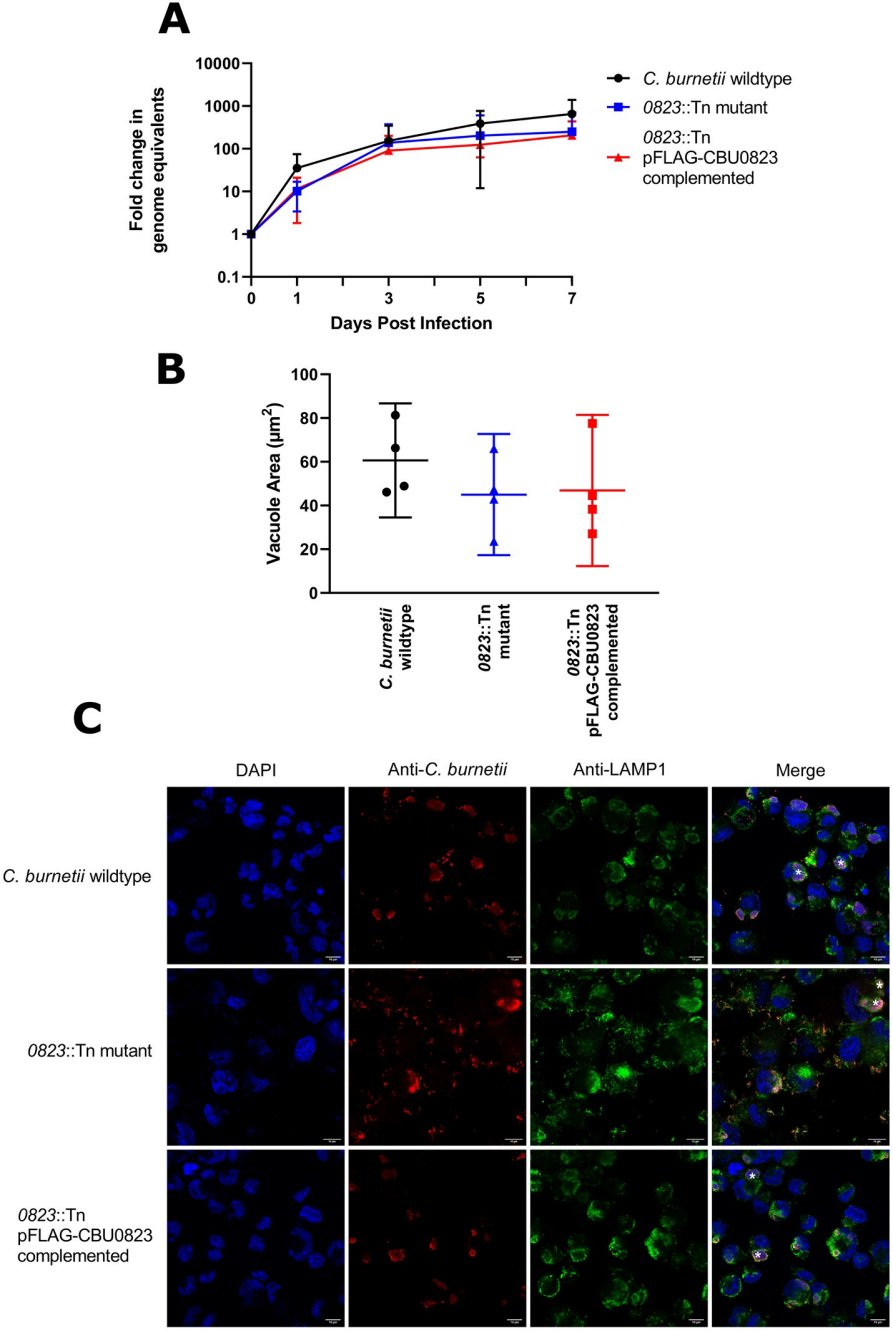
*cbu0823* is not required for efficient intracellular replication of *C*. *burnetii* in THP-1 cells. Intracellular replication within THP-1 macrophage-like cells of *C*. *burnetii* wildtype, *0823*::Tn mutant and *0823*::Tn pFLAG-CBU0823 complemented mutant strains over 7 days depicted in fold change of genome equivalents (GE) measured by qPCR for *ompA*. (A) Fold change in GE was plotted over 7 days as mean with error bars representing standard deviation from six independent biological replicates. There was no difference in replication between strains (*p* > 0.05). (B) Average vacuole area, measured in μm^2^ on representative immunofluorescent images of each strain and plotted as mean with error bars representing standard deviation for four of the six replicates. There was no significant difference between the three strains (*p* > 0.05). (C) Representative confocal immunofluorescent micrographs of THP-1 cells 3 days post-infection with *C*. *burnetii* wildtype, *0823*::Tn mutant and *0823*::Tn complemented mutant strains. There were no significant differences in CCV size and organism numbers between strains. Anti-*Coxiella* antibody (red), anti-LAMP-1 antibody (green) and DAPI (blue) were used to stain cells and white asterisks indicate CCVs. The scale bar depicts 10 μm.

Finally, we investigated the impact what impact the metabolic alterations seen in the *0823*::Tn mutant might have on *C*. *burnetii* replication inside host cells. The fold change in genome equivalents of *C*. *burnetii* strains was measured within THP-1 macrophage-like cells infected with an MOI of 25 over 7 days by qPCR. No significant differences were observed in replication between strains over 7 days (*p* > 0.05) (Fig 9A). The strains were visibly indistinguishable on representative immunofluorescent images taken on day 3 post-infection (Fig 9C) and no significant differences in vacuole size were observed (Fig 9B).

## Discussion

It is yet to be determined how *C*. *burnetii* accomplishes lactate synthesis, though it can reproducibly incorporate stable isotope label into lactate in multiple studies [6,7]. This study has demonstrated that CBU1241 and CBU0823 are unlikely to be responsible for the lactate production observed. Neither CBU0823 nor CBU1241 exhibited any LDH activity in *in vitro* enzyme activity assays and CBU0823 did not produce lactate from an MLE reaction. *In vivo* assessment indicated the *0823*::Tn mutant was as able to incorporate ^13^C label into lactate in an equivalent manner to wildtype. Taken together, these results indicate that the metabolic pathway by which *C*. *burnetii* synthesizes lactate is still to be established.

The characterization in this study of CBU1241 revealed robust OAA reduction. *in vitro* CBU1241 is clearly affected by substrate inhibition, with activity diminishing above 0.6 mM OAA. 0.6 mM is a lower concentration than for many other published bacterial MDHs, though it is comparable to pig heart mitochondrial MDH and higher than some, such as *T*. *flavus* MDH [61,71,72]. Due to its instability, accurate intracellular OAA values are currently not available. Together with the lack of knowledge regarding the MDH malate reduction reaction, the level of substrate inhibition CBU1241 experiences *in vivo* remains unknown.

The variation of two key residues within CBU0823 from other ME suggested the enzyme could possess the substrate flexibility characteristic of malic enzymes (MEs) [37]. In this study, the *in vitro* experiments demonstrated recombinant CBU0823 lacked any detectable LDH or MLE function but did possess both ME and MDH activity. Nonetheless, the simple *in vitro* assessment of CBU0823, and CBU1241, function may not fully represent enzyme capabilities *in vivo*.

The reduced incorporation of label into metabolites across central carbon metabolism in the *0823*::Tn mutant cultures appears similar to the rerouting of carbon away from glycolysis and the TCA cycle noted in ME mutant strains of *Sinorhizobium meliloti*, a soil dwelling symbiont bacteria associated with alfalfa roots [73]. These metabolism modifications may be attributable to the loss of the cataplerotic reaction catalyzed by MEs, removing malate from the TCA cycle, and the resultant anaplerotic reaction of pyruvate conversion to acetyl CoA for entry into the TCA cycle [74,75]. Instead, acetyl CoA replenishment from TCA cycle intermediates would require the anaplerotic reaction of OAA to phosphoenolpyruvate (PEP) by PEP carboxykinase (PckA) [76,77]. In other words, TCA cycle function in the ME mutants likely would rely on the slowest reaction in the cycle, the MDH enzyme, to produce sufficient OAA to both remain in the TCA cycle and to exit the TCA cycle for replenishment of acetyl CoA. From this data, we cannot determine how the loss of the secondary MDH function of CBU0823 in the *0823*::Tn mutant contributed to TCA cycle dysfunction.

The replication of the *0823*::Tn mutant was not lower than wildtype intracellularly. The *S*. *meliloti* ME mutants with comparable metabolome changes did exhibit slowed replication, in contrast to *E*. *coli* ME mutants where simultaneous removal of the alternate pyruvate producing PckA pathway was required to affect replication [73,78]. The MDH-PckA pathway may provide sufficient OAA and PEP to maintain typical *C*. *burnetii* replication, especially as the normal replication rate of *C*. *burnetii* is comparatively slow [77]. Furthermore, reduction in TCA cycle activity reduces oxidative stress, an issue for *C*. *burnetii* within its replicative niche [6,70], and the reduced TCA activity may be of benefit to the *0823*::Tn mutant. Moreover, this study concentrates on glucose utilization whereas *C*. *burnetii* has a metabolism capable of utilizing amino acids, thus the additional energy required by the *0823*::Tn mutant for replication could be provided by carbon sources other than glucose [7,79].

This study has demonstrated the *C*. *burnetii* gene *cbu1241* encodes an enzyme with *in vitro* MDH function and *cbu0823* encodes an enzyme with both ME and MDH function *in vitro*, albeit a less efficient MDH than *cbu1241*. Neither enzyme demonstrated the capacity to produce lactate *in vitro*, and *in vitro* labelling studies suggested that CBU1241 was not responsible for lactate production *in vivo*. Although not required for efficient replication in axenic culture or within host cells, *cbu0823*, most likely through its malic enzyme function, is required for normal glycolysis and TCA cycle function in *C*. *burnetii*. Future work examining lactate production by *C*. *burnetii* may include large scale screening studies of mutant libraries and/or sophisticated bioinformatic analysis, in order to identify new potential candidate enzymes.

## Supporting information

S1 Fig Protein sequence alignments of representative (A) MDHs and LDHs including CBU1241, and (B) MEs and MLEs including CBU0823. The pertinent accession numbers are provided in parentheses. Residues in sequences sharing identity with consensus are highlighted.
(PDF)

S2 Fig Protein gels and corresponding western blots of purified recombinant proteins. (A) Protein gel (left) and corresponding western blot (right) probed for GST showing purified 6 μg GST-CBU1241 and 2 μg GST proteins of approximately expected size, 61.5 kDa and 26 kDa respectively. (B) Protein gel (left) and corresponding western blot (right) probed for 6xHis-tag showing purified 4 μg 6xHis-CBU0823 and 1 μg 6xHis-oMLE, each of the approximately expected sizes of 63.5 kDa and 62.3 kDa respectively, and the lack of detectable 6xHis-tagged product in the mock purification negative control. (C) Western blot probed for 3xFLAG of *C*. *burnetii 0823*::Tn pFLAG-CBU0823 whole cell lysate showing *C*. *burnetii* expressing a protein product of approximately expected size, 64.5 kDa, from the complementation plasmid. The marker shown is from the corresponding stain-free protein gel.
(TIF)

S3 Fig Lineweaver-Burk (A, C, E, G) and reciprocal of the quotient velocity (B, D, F, H) plots. GST-CBU1241 in relation to OAA (A and B) from Michaelis-Menten data presented in Fig 4A. 6xHis-CBU0823 in relation to malate (C and D) from Michaelis-Menten data presented in Fig 6A, to NAD^+^ (E and F) from Michaelis-Menten data presented in Fig 6B, and to MnCl_2_ (G and H) from Michaelis-Menten data presented in Fig 6C. Error bars represent standard deviation around the mean from at least three independent experiments.
(TIF)

S4 Fig *In vitro* 6xHis-oMLE activity assays for ME, LDH and MDH function, using the oMLE storage buffer as assay buffer. (A) ME activity was observed for 6xHis-oMLE in a ME activity assay containing 10 mM malate, 1 mM NAD^+^ and 1 mM. Omission of MnCl_2_ reduced activity. (B) MDH activity was observed for 6xHis-oMLE in an assay containing 5 mM malate and 0.5 mM NADH. Activity was greatly increased by the addition of 1 mM MnCl_2_. (D) LDH activity was exhibited by 6xHis-oMLE in an LDH assay containing 5 mM pyruvate and 0.5 mM NADH. Activity was unchanged by the addition of 1 mM MnCl_2_. All assays used 28.5 μg 6xHis-oMLE per well or the equivalent volume of 6xHis-mock purification negative control and were performed at 45°C in 100 mM HEPES/0.1 mMnCl_2_ pH 6.0 buffer. Activity was measured using increase or decrease of light absorbance at 340 nm, correlating to NADH oxidation and NAD^+^ reduction respectively during enzyme activity. Error bars represent standard deviation around the mean from at least three independent experiments.
(TIF)

S5 Fig Isotopologue distribution patterns for metabolites in or directly related to the TCA cycle of *C*. *burnetii* wildtype, *0823*::Tn mutant and *0823*::Tn pFLAG-CBU0823 complemented strains. *C*. *burnetii* incubated with [^13^C]glucose supplemented ACCM-2 for 7 days before GC-MS analysis to determine ^13^C incorporated into the extracted metabolites. Mean isotopologue distribution of each metabolite detected, corrected for natural isotopic abundance with 0% unlabelled biomass, was graphed using DExSI for each strain. Error bars represent standard deviation from five biological replicates. Solid lines represent a single enzyme and dotted lines represent multiple enzymes. The blue line denotes the activity of CBU0823 as a ME. The green line denotes CBU1241 as an MDH. The orange line represents an unestablished conversion of pyruvate to lactate by an LDH-type reaction. PEP = phosphoenolpyruvic acid.
(TIF)

S1 Table PCR oligonucleotides used in this study, with restriction enzyme sites underlined. (DOCX)

S2 Table Protein sequence identity (%) for representative (A) MDHs and LDHs compared to CBU1241 and (B) MEs and MLEs compared to CBU0823. CBU1241 and CBU0823 are highlighted in yellow and notable identities described in the text are highlighted in green. The pertinent accession numbers are provided in parenthesis.
(DOCX)

S1 Raw images (TIF)
