# Supplementary material for: Characterisation of putative lactate synthetic pathways of Coxiella burnetii
Source: PLoS One. 2021 Aug 13;16(8):e0255925. doi: 10.1371/journal.pone.0255925 (PMC8362950; doi:10.1371/journal.pone.0255925)
Supplement: S1 Fig — The pertinent accession numbers are provided in parentheses. Residues in sequences sharing identity with consensus are highlighted. (PDF) [file pone.0255925.s001.pdf]

A

```
Consensus                              MXXXXXXXXXXXXXXXXXXXXXXXXXXXXXXXXXXXXXXXXXXXXXLXXXXXXXXXXXXX   60
E. coli L-LDH (P33232)                 MIISAASDYRAAAQRILPPFLFHYMDGGAYSEYTLRRNVEDLSEVALRQRILKNMSDLSL   60
Pig heart cytop. MDH (P11708)          ------------------------------------------------------------
T. thermophilus MDH (AFH39418.1)       ------------------------------------------------------------
L. pneumophila MDH (Q5ZT13.1)          ------------------------------------------------------------
C. burnetii Cbu1241 (NP_820236.1)      ------------------------------------------------------------
Pig heart mitoch. MDH (P00346.2)       ML-S-----------------------------------------ALARPAGAAL-----   13
E. coli MDH (NP_417703.1)              ------------------------------------------------------------
B. melitensis LDH/MDH (3GVH_B)         ------------------------------------------------------------
Cow heart LDH (AAI42007.1)             -M-A-----------------------------------------TLKEKLIAPV-----   12
L. casei LDH (2ZQY_A)                  ------------------------------------------------------------

Consensus                              XXXXXXXXXXXXXXXXXXGXXGXXGXXXX-----XXXXXXXXXXXXXXXXXXLXXXXXXX  115
E. coli L-LDH (P33232)                 ETTLFNEKLSMPVALAPVGLCGMYARRGEVQAAKAADAHGIPFTLSTVSVCPIEEVAPAI  120
Pig heart cytop. MDH (P11708)          --------MSEPIRVLVTGAAGQIAYSLL-----YSIGNGSVFGKDQPIILVLLDITPMM   47
T. thermophilus MDH (AFH39418.1)       --------MKAPVRVAVTGAAGQIGYSLL-----FRIAAGEMLGKDQPVILQLLEIPQAM   47
L. pneumophila MDH (Q5ZT13.1)          -------MTNNRVRVAVTGAAGQIGYALV-----FRIASGQMFGPNTEVELNLLELEPAL   48
C. burnetii Cbu1241 (NP_820236.1)      --------MAKHVKVAVTGAAGQIGYALL-----FRLASGQAFGLDTTVDLHLLEIEPAL   47
Pig heart mitoch. MDH (P00346.2)       -RRSFSTSXQNNAKVAVLGASGGIGQPLS-----LLLKN-SPLV----SRLTLYDIA---   59
E. coli MDH (NP_417703.1)              ------------MKVAVLGAAGGIGQALA-----LLLKTQLPSG----SELSLYDIAP--   37
B. melitensis LDH/MDH (3GVH_B)         -----GPGSMARNKIALIG-SGMIGGTLA-----HLAGL-KEL-----GDVVLFDIAE--   41
Cow heart LDH (AAI42007.1)             ---AEEETRIPNNKITVVG-VGQVGMACA-----ISILG-KSLT----DELALVDVLE--   56
L. casei LDH (2ZQY_A)                  ---MASITDKDHQKVILVG-DGAVGSSYA-----YAMVL-QGIA----QEIGIVDIFK--   44

Consensus                              XXXXGXXXXLXXXXXXXXXXXXXXXXXXXXXXXXXBXXXXXXXXPXXXGXXRXDLXXX--  173
E. coli L-LDH (P33232)                 KRP------MWFQLYVLRDRGFMRNALERAKAAGCSTLVFTVDMPTPGARYRDAHSGMSG  174
Pig heart cytop. MDH (P11708)          GVLDGVLMELQDCALPL-LKDVIATDKEEIAFKDLDVAILVGSMPRRDGMERKDLLKA--  104
T. thermophilus MDH (AFH39418.1)       KALEGVVMELEDCAFPL-LAGLEATDDPKVAFKDADYALLVGAAPRKAGMERRDLLEM--  104
L. pneumophila MDH (Q5ZT13.1)          PSLEGVAMELDDCAFPL-LKRIVCTADLNKAMDGVNWALLVGSVPRKQGMERSDLLQI--  105
C. burnetii Cbu1241 (NP_820236.1)      PALKGVVMELEDCAFPL-LRNMVVTSDPRVAFNDVNWALLVGAAPRKAGMERKDLLEK--  104
Pig heart mitoch. MDH (P00346.2)       HT-PGVAADLSHIETRATVKGYLGPEQLPDCLKGCDVVVIPAGVPRKPGMTRDDLFNT--  116
E. coli MDH (NP_417703.1)              VT-PGVAVDLSHIPTAVKIKGFSGEDATP-ALEGADVVLISAGVARKPGMDRSDLFNV--   93
B. melitensis LDH/MDH (3GVH_B)         GTPQGKGLDIAESSPVDGFDAKFTGANDYAAIEGADVVIVTAGVPRKPGMSRDDLLGI--   99
Cow heart LDH (AAI42007.1)             DKLKGEMMDLQHGSLFLQ-TPKIVADKDYSVTANSKIVVVTAGVRQQEGESRLNLVQR--  113
L. casei LDH (2ZQY_A)                  DKTKGDAIDLSNALPF-T-SPKKIYSAEYSDAKDADLVVITAGAPQKPGETRLDLVNK--  100

Consensus                              -NXXXXXXXXXXXXXXXXXXXXXXXXXNPXBXXXXXX---XXXXXXXXXXXXXXXXXXLD  229
E. coli L-LDH (P33232)                 PNAAMRRYL-QAVTH-PQWAWDVGLNGRPHDL-----------------------------  204
Pig heart cytop. MDH (P11708)          -NVKIFKCQGAALDKYAKKSVKVIVVGNPANTNCLTA---SKSAPSIPKENFSC-LTRLD  159
T. thermophilus MDH (AFH39418.1)       -NGKIFTEQGRALAEVAKKDVKVLVVGNPANTNALIA---YKNAPGLNPRNFTA-MTRLD  159
L. pneumophila MDH (Q5ZT13.1)          -NGGIFTKQGQAINDYASDDVRVFVVGNPCNTNCLIA---MNHAKDVPSDRFYA-MTTLD  160
C. burnetii Cbu1241 (NP_820236.1)      -NGSIFAGQGKAINENAASDVRIFVVGNPCNTNCLIA---MNNAPDIPKDRFYA-MTRLD  159
Pig heart mitoch. MDH (P00346.2)       -NATIVATLTAACAQHCPDA-MICIISNPVNSTIPITAEVFKKHGVYNPNKIFG-VTTLD  173
E. coli MDH (NP_417703.1)              -NAGIVKNLVQQVAKTCPKA-CIGIITNPVNTTVAIAAEVLKKAGVYDKNKLFG-VTTLD  150
B. melitensis LDH/MDH (3GVH_B)         -NLKVMEQVGAGIKKYAPEA-FVICITNPLDAMVWAL---QKFSG-LPAHKVVGMAGVLD  153
Cow heart LDH (AAI42007.1)             -NVNVFKFIIPQIVKYSPDC-IIIVVSNPVDILTYVT---WKLSG-LPKHRVIGSGCNLD  167
L. casei LDH (2ZQY_A)                  -NLKILKSIVDPIVDSGFNG-IFLVAANPVDILTYAT---WKLSG-FPKNRVVGSGTSLD  154

Consensus                              XXRXXXXXAXXXXXXXXXXXXXXXXXXXXXXXXXPXXXXXXXXXX----XXXXXXXXXXX  285
E. coli L-LDH (P33232)                 -GNISAYLGK-----PTGLED-----------YIGWLGNNFDPS-ISWKDLEWIR-DFWD  245
Pig heart cytop. MDH (P11708)          HNRAKAQIALKLGVTSDDVKNVIIWGNHSSTQYPDVNHAKVKLQAKEVGVYEAVKDDSWL  219
T. thermophilus MDH (AFH39418.1)       HNRAKAQLAKKTGVPVDRIRRMTVWGNHSSTMFPDLFHAEVDGR----PALELV-DMEWY  214
L. pneumophila MDH (Q5ZT13.1)          ELRARTQLAKKAGVDITAVTQMTIWGNHSATQYPDFYNAKINGT----SAAQVINDETWL  216
C. burnetii Cbu1241 (NP_820236.1)      QNRAIGQLALKAGVDVPSVKNMIIWGNHSSTQYPDFYHATIDGK----PATEVIRDKNWL  215
Pig heart mitoch. MDH (P00346.2)       IVRANAFVAELKGLDPARVSVPVIGGHAGKTIIPLISQCT-PKV----DF----------  218
E. coli MDH (NP_417703.1)              IIRSNTFVAELKGKQPGEVEVPVIGGHSGVTILPLLSQV--PGV----SF----------  194
B. melitensis LDH/MDH (3GVH_B)         SARFRYFLSEEFNVSVEDVTVFVLGGHGD-SMVPLARYSTVAGI----PLPDLVK-MGWT  207
Cow heart LDH (AAI42007.1)             SARFRYLMAEKLGIHPSSCHGWILGEHGD-SSVAVWSGVNVAGV----SLQELNP-EMGT  221
L. casei LDH (2ZQY_A)                  TARFRQSIAEMVNVDARSVHAYIMGEHGD-TEFPVWSHANIGGV----TIAEWVK-AHPE  208

Consensus                              --XXXXXXXXXXX----------XXXXXXXXXXX-XXXXXXXXXXXXXXXXXXXXXXX  332
E. coli L-LDH (P33232)                 ------GPMVIKGILDPEDARDAVRFGADGIVVSNH-GG--------RQLDGVLSSARAL  290
Pig heart cytop. MDH (P11708)          ------KGEFITTV----------QQRGAAVIKARK-LSSAMSAAKAICDHVRDIWFGT  261
T. thermophilus MDH (AFH39418.1)       ------EKVFIPTV----------AQRGAAIIQARG-ASSAASAANAAIEHIRDWALGT  256
L. pneumophila MDH (Q5ZT13.1)          ------KETFVSTV----------QQRGAAVIKARG-SSSAASAANAIITGVNHLVTDT  258
C. burnetii Cbu1241 (NP_820236.1)      ------LNDFISVI----------QQRGAAVIKARG-ASSAASAANAALDSVWSLINTT  257
Pig heart mitoch. MDH (P00346.2)       --PQDQLSTLTGRI----------QEAGTEVVKAKAGAGSATLSMAYAGARFVFSLVDAM  266
E. coli MDH (NP_417703.1)              --TEQEVADLTKRI----------QNAGTEVVEAKAGGGSATLSMGQAAARFGLSLVRAL  242
B. melitensis LDH/MDH (3GVH_B)         --SQDKLDKIIQRT----------RDGGAEIVGLLK-TGSAFYAPAASAIQMAESYLKDK  254
Cow heart LDH (AAI42007.1)             DNDSENWKEVHKMV----------VESAYEVIKL---KGYTNWAIGLSVADLIESMLKNL  268
L. casei LDH (2ZQY_A)                  I-KEDKLVKMFEDV----------RDAAYEIIKL---KGATFYGIATALARISKAILNDE  254

Consensus                              XXXXXXXXXXXXXG------XXGXXXXX------------------XXXXPXXXXXXGXX  368
E. coli L-LDH (P33232)                 PAIAD-----AVKGDIAILADSGIRNGLDVVRMIALGADTVLLGRAFLYALATAGQAGVA  345
Pig heart cytop. MDH (P11708)          PEGEFVSMGIISDG-----NSYGVPDDL------------------LYSFPVTIKD-KTW  297
T. thermophilus MDH (AFH39418.1)       PEGDWVSMAVPSQG------EYGIPEGI------------------VYSFPVTAKD-GAY  291
L. pneumophila MDH (Q5ZT13.1)          PAGESFSMCRRSKG------EYGVDEGL------------------IFSFPCRREH-GEL  293
C. burnetii Cbu1241 (NP_820236.1)      PADDNYSVALCAQG------QYGVDEGL------------------IFSFPCRTEN-GVV  292
Pig heart mitoch. MDH (P00346.2)       NGKEGVVECSFVKS------QE--TDCP------------------YFSTPLLLGKKGIE  300
E. coli MDH (NP_417703.1)              QGEQGVVECAYVEG------DG--QYAR------------------FFSQPLLLGKNGVE  276
B. melitensis LDH/MDH (3GVH_B)         KRVLP--VAAQLSG------QYGVK-DM------------------YVGVPTVIGANGVE  287
Cow heart LDH (AAI42007.1)             SRIHP--VSTMVKG------MYGIENEV------------------FLSLPCILNARGLT  302
L. casei LDH (2ZQY_A)                  NAVLP--LSVYMDG------QYGLN-DI------------------YIGTPAVINRNGIQ  287

Consensus                              XXXXXXXXXXXXXXXXXXXXXLXXXXXX--XXXXXXXXXXXXXPMAKGNAA          418
E. coli L-LDH (P33232)                 NLLNLIEKEM-KVAMTLTGAKSISEITQDSLVQGLGKELPAALAPMAKGNAA          396
Pig heart cytop. MDH (P11708)          KIVEGLPINDFSREKMDLTAKELAEEKET--AFEFLSSA-------------          334
T. thermophilus MDH (AFH39418.1)       QVVEGLEINEFARKRMELTAQELLDEMEQ--VKALGLI--------------          327
L. pneumophila MDH (Q5ZT13.1)          KVVENLEFNDFGRERFNTTLNELRSERDT--VKSLGLLD-------------          330
C. burnetii Cbu1241 (NP_820236.1)      SVIEEIEHNEFGQQKLKETLDELREERDA--VEALGLI--------------          328
Pig heart mitoch. MDH (P00346.2)       KNLGIGKISPFEEKMIAEAIPELKASI------KKGEEFVKNMK--------          338
E. coli MDH (NP_417703.1)              ERKSIGTLSAFEQNALEGMLDTLKKDI------ALGEEFVNK----------          312
B. melitensis LDH/MDH (3GVH_B)         RIIEIDLDKD-EKAQFDKSVASVAGLCEA--CIGIAPSLK------------          324
Cow heart LDH (AAI42007.1)             SVINQKLKDE-EVAQLKKSADTLWGIQKD--LKDL-----------------          334
L. casei LDH (2ZQY_A)                  NILEIPLTDH-EEESMQKSASQLKKVLTD--AFAKNDIETRQ----------          326
```

B

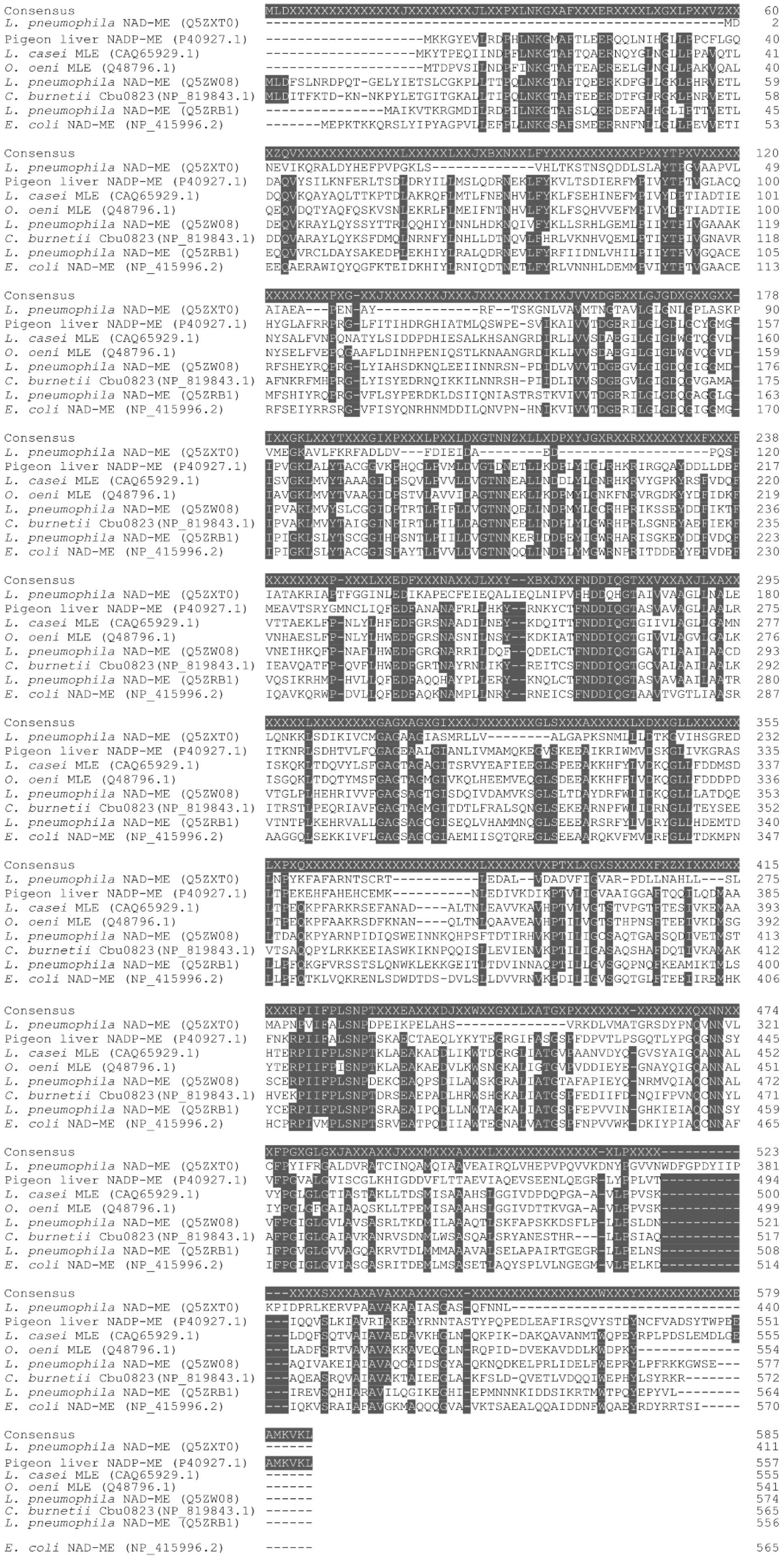
Consensus
L. pneumophila NAD-ME (Q5ZXT0)
Pigeon liver NADP-ME (P40927.1)
L. casei MLE (CAQ65929.1)
O. oeni MLE (Q48796.1)
L. pneumophila NAD-ME (Q5ZW08)
C. burnetii Cbu0823(NP_819843.1)
L. pneumophila NAD-ME (Q5ZRB1)
E. coli NAD-ME (NP_415996.2)

S1 Fig. Protein sequence alignments of representative (A) MDHs and LDHs including CBU1241, and (B) MEs and MLEs including CBU0823.

The pertinent accession numbers are provided in parentheses. Residues in sequences sharing identity with consensus are highlighted.
