# Supplementary figures and images for: Characterisation of putative lactate synthetic pathways of Coxiella burnetii
Source: PLoS One. 2021 Aug 13;16(8):e0255925. doi: 10.1371/journal.pone.0255925 (PMC8362950; doi:10.1371/journal.pone.0255925)

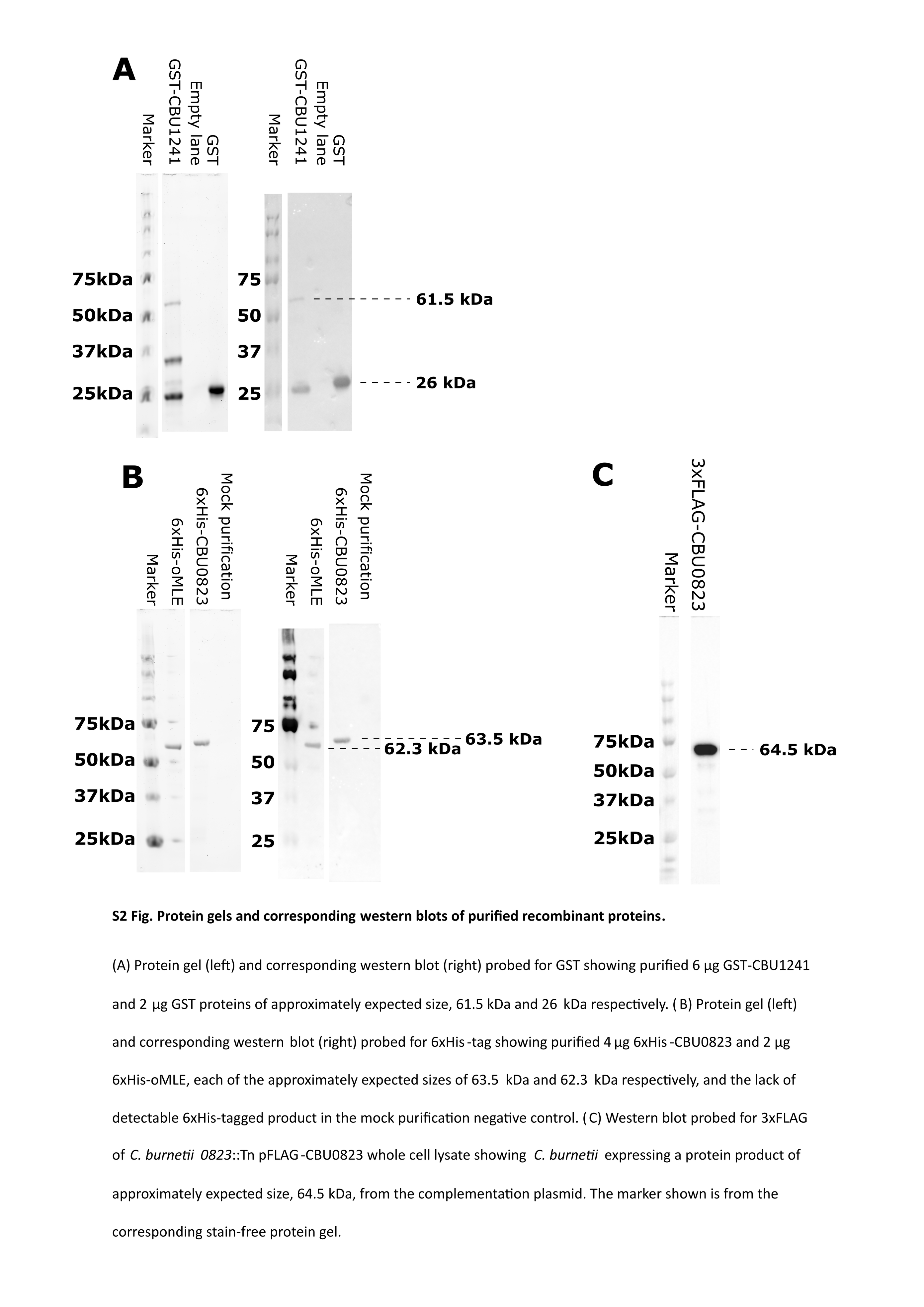

Supplement: S2 Fig — (A) Protein gel (left) and corresponding western blot (right) probed for GST showing purified 6 μg GST-CBU1241 and 2 μg GST proteins of approximately expected size, 61.5 kDa and 26 kDa respectively. (B) Protein gel (left) and corresponding western blot (right) probed for 6xHis-tag showing purified 4 μg 6xHis-CBU0823 and 1 μg 6xHis-oMLE, each of the approximately expected sizes of 63.5 kDa and 62.3 kDa respectively, and the lack of detectable 6xHis-tagged product in the mock purification negative control. (C) Western blot probed for 3xFLAG of C. burnetii 0823::Tn pFLAG-CBU0823 whole cell lysate showing C. burnetii expressing a protein product of approximately expected size, 64.5 kDa, from the complementation plasmid. The marker shown is from the corresponding stain-free protein gel. (TIF) [file pone.0255925.s002.tif]

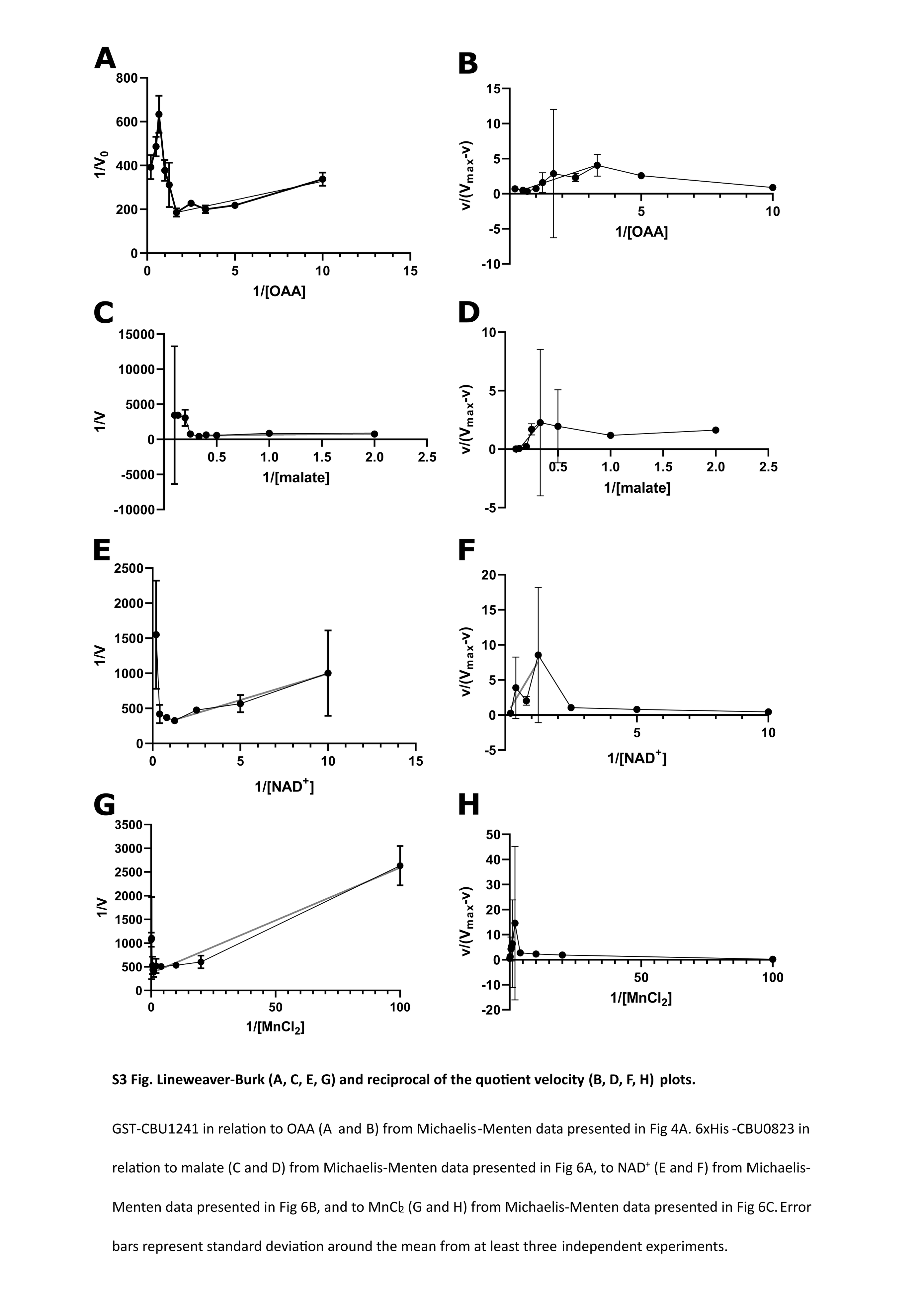

Supplement: S3 Fig — GST-CBU1241 in relation to OAA (A and B) from Michaelis-Menten data presented in Fig 4A. 6xHis-CBU0823 in relation to malate (C and D) from Michaelis-Menten data presented in Fig 6A, to NAD+ (E and F) from Michaelis-Menten data presented in Fig 6B, and to MnCl2 (G and H) from Michaelis-Menten data presented in Fig 6C. Error bars represent standard deviation around the mean from at least three independent experiments. (TIF) [file pone.0255925.s003.tif]

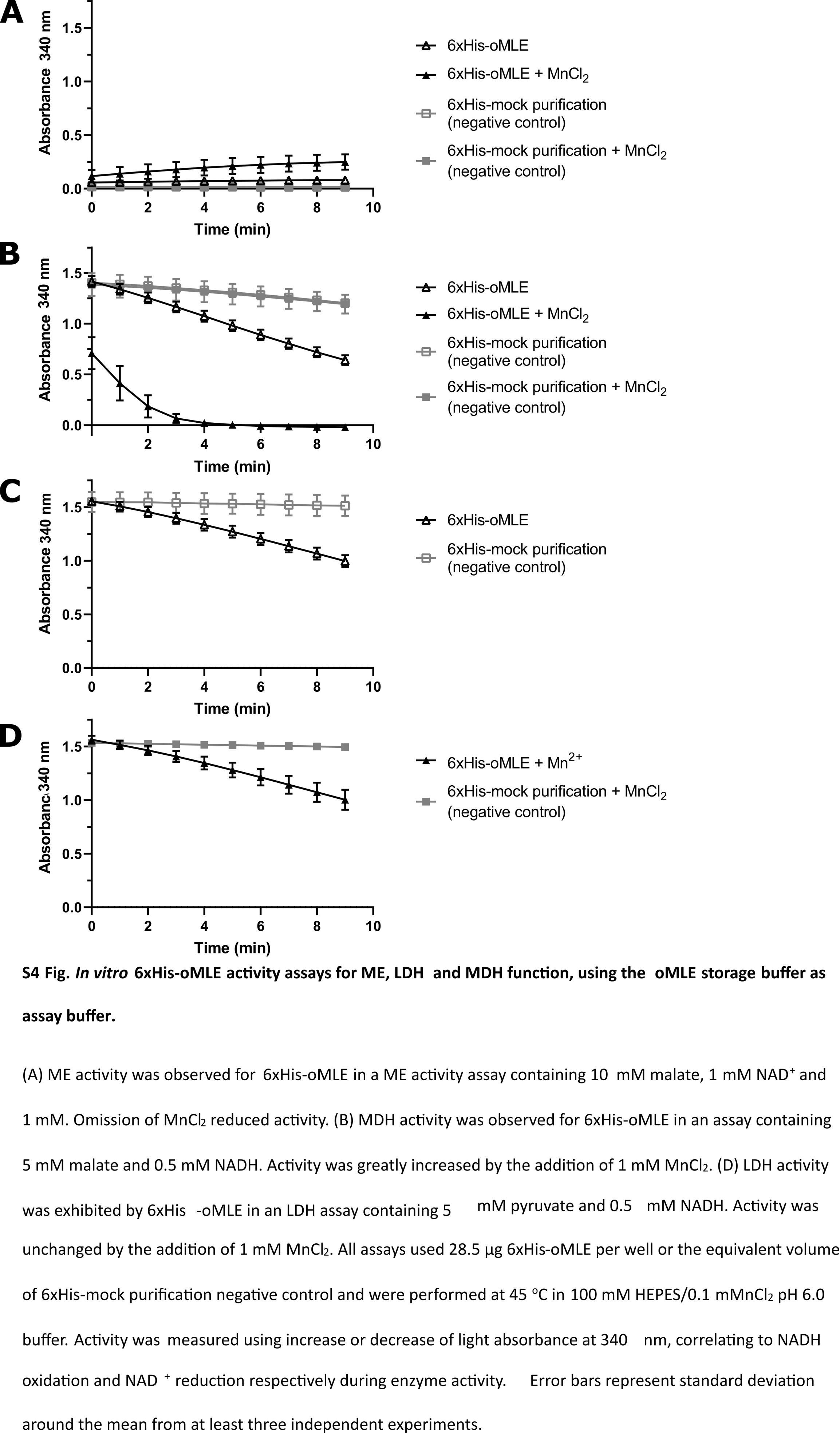

Supplement: S4 Fig — (A) ME activity was observed for 6xHis-oMLE in a ME activity assay containing 10 mM malate, 1 mM NAD+ and 1 mM. Omission of MnCl2 reduced activity. (B) MDH activity was observed for 6xHis-oMLE in an assay containing 5 mM malate and 0.5 mM NADH. Activity was greatly increased by the addition of 1 mM MnCl2. (D) LDH activity was exhibited by 6xHis-oMLE in an LDH assay containing 5 mM pyruvate and 0.5 mM NADH. Activity was unchanged by the addition of 1 mM MnCl2. All assays used 28.5 μg 6xHis-oMLE per well or the equivalent volume of 6xHis-mock purification negative control and were performed at 45°C in 100 mM HEPES/0.1 mMnCl2 pH 6.0 buffer. Activity was measured using increase or decrease of light absorbance at 340 nm, correlating to NADH oxidation and NAD+ reduction respectively during enzyme activity. Error bars represent standard deviation around the mean from at least three independent experiments. (TIF) [file pone.0255925.s004.tif]

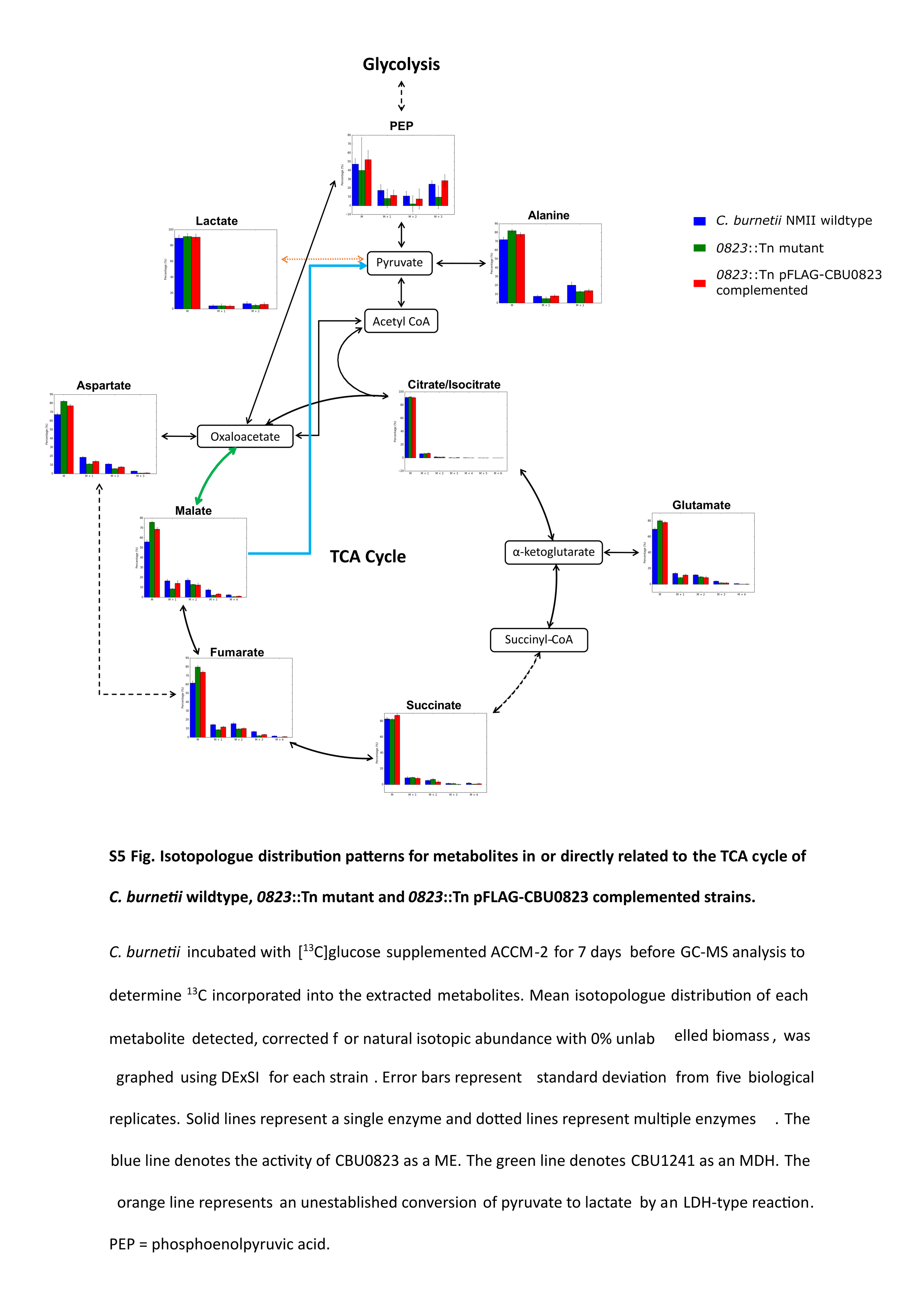

Supplement: S5 Fig — C. burnetii incubated with [13C]glucose supplemented ACCM-2 for 7 days before GC-MS analysis to determine 13C incorporated into the extracted metabolites. Mean isotopologue distribution of each metabolite detected, corrected for natural isotopic abundance with 0% unlabelled biomass, was graphed using DExSI for each strain. Error bars represent standard deviation from five biological replicates. Solid lines represent a single enzyme and dotted lines represent multiple enzymes. The blue line denotes the activity of CBU0823 as a ME. The green line denotes CBU1241 as an MDH. The orange line represents an unestablished conversion of pyruvate to lactate by an LDH-type reaction. PEP = phosphoenolpyruvic acid. (TIF) [file pone.0255925.s005.tif]

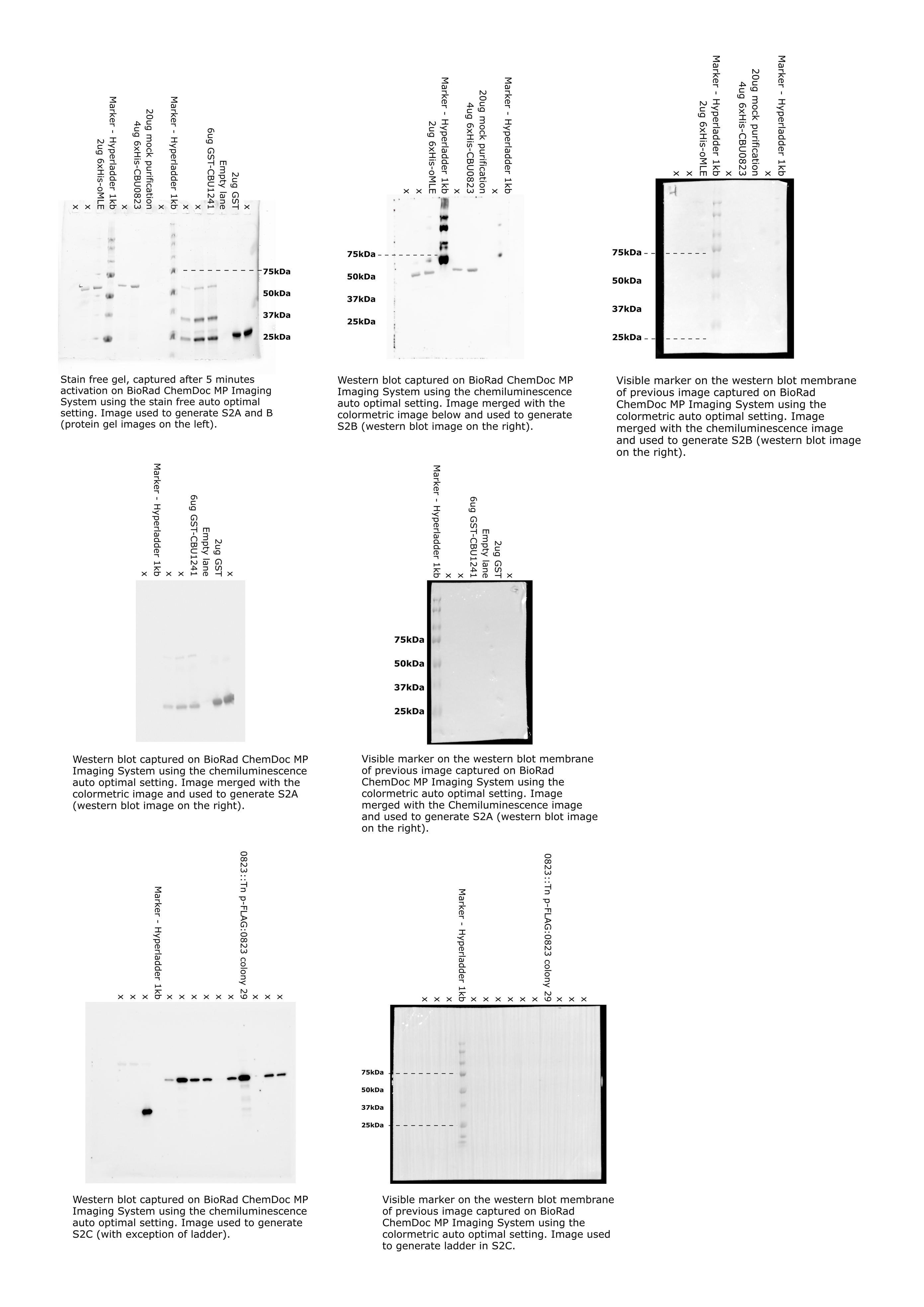

Supplement: S1 Raw images — (TIF) [file pone.0255925.s008.tif]
