# Supplementary material for: Characterisation of putative lactate synthetic pathways of Coxiella burnetii
Source: PLoS One. 2021 Aug 13;16(8):e0255925. doi: 10.1371/journal.pone.0255925 (PMC8362950; doi:10.1371/journal.pone.0255925)
Supplement: S1 Table — (DOCX) [file pone.0255925.s006.docx]

**S1 Table. PCR oligonucleotides used in this study, with restriction enzyme sites underlined.**

| **Name** | **Sequence (5’ to 3’)** | **Annealing temperature used (^o^C)** | **Purpose** |
| --- | --- | --- | --- |
| *cbu0823* F BamHI | CGCGGATCCATGCTAGACATTACCTTC | 52 | Cloning into pQE-30 (protein expression) and pJB-3xFLAG (complementation) |
| *cbu0823* R SalI | ACGCGTCGACTTATCTTTTTCGGTAAGAT | 52 | Cloning into pQE-30 (protein expression) |
| *cbu0823* R NotI | AAGGAAAAAAGCGGCCGCTTATCTTTTTCGGTAAGATAA | 52 | Cloning into pJB-3xFLAG (complementation) |
| *cbu1241* F BamHI | CGCGGATCCATGGCTAAGCATGTCAAAGTAGC | 52 | Cloning into pGEX-4-T1 (protein expression) |
| *cbu1241* R SalI | ACGCGTCGACTTAAATTAATCCCAACGCCTC | 52 | Cloning into pGEX-4-T1 (protein expression) |
| *O. oeni MLE* F | CGCGGATCCATGACAGATCCAGTAAGTATT | 52 | Cloning into pQE-30 (protein expression) |
| *O. oeni MLE* R | CGCCTGCAGTTAGTATTTCGGCTCCCAC | 52 | Cloning into pQE-30 (protein expression) |
| pQE-30 F | CGGATAACAATTTCACACAG | 50 | Sequencing pQE-30:*cbu0823* construct |
| pQE-30 R | GTTCTGAGGTCATTACTGG | 50 | Sequencing pQE-30:*cbu0823* construct |
| pGEX-4-T1 F | GGGCTGGCAAGCCACGTTTGGTG | 50 | Sequencing pGEX-4-T1:*cbu1241* construct |
| pGEX-4-T1 R | CCGGGAGCTGCATGTGTCAGAGG | 50 | Sequencing pGEX-4-T1:*cbu1241* construct |
| pJB-3xFLAG F | GAGCTGTTGACAATTAATCATC | 50 | Sequencing pFLAG-CBU0823 complementation construct |
| pJB-3xFLAG R | GGATTCATCGACTGTGGCCG | 50 | Sequencing pFLAG-CBU0823 complementation construct |
| 5171 | CTTTTTACGGTTCCTGGGCTT | 52 | Transposon detection |
| 5174 | TGGCGATTCAGGTTCATCATG | 52 | Transposon detection |
| *ompA* F | CAGAGCCGGGAGTCAAGCT | 60 | *Coxiella* genome quantification [51] |
| *ompA* R | CTGAGTAGGAGATTTGAATCGC | 60 | *Coxiella* genome quantification [51] |
