# Supplementary material for: Characterisation of putative lactate synthetic pathways of Coxiella burnetii
Source: PLoS One. 2021 Aug 13;16(8):e0255925. doi: 10.1371/journal.pone.0255925 (PMC8362950; doi:10.1371/journal.pone.0255925)
Supplement: S2 Table — CBU1241 and CBU0823 are highlighted in yellow and notable identities described in the text are highlighted in green. The pertinent accession numbers are provided in parenthesis. (DOCX) [file pone.0255925.s007.docx]

**S2 Table. Protein sequence identity (%) for representative (A) MDHs and LDHs compared to CBU1241 and (B) MEs and MLEs compared to CBU0823.** CBU1241 and CBU0823 are highlighted in yellow and notable identities described in the text are highlighted in green. The pertinent accession numbers are provided in parenthesis.

**(A)**

|  | *Escherichia coli*  L-LDH (P33232) | Pig heart cytoplasmic MDH (P11708) | *Thermus thermophilus* MDH (AFH39418.1) | *Legionella pneumophila* MDH (Q5ZT13.1) | *Coxiella burnetii* CBU1241 (NP_820236.1) | Pig heart mitochondrial MDH (P00346.2) | *E. coli* MDH (NP_417703.1) | *Brucella melitensis* LDH/MDH (3GVH_B) | Cow heart LDH (AAI42007.1) | *Lactobacillus casei* LDH (2ZQY_A) |
| --- | --- | --- | --- | --- | --- | --- | --- | --- | --- | --- |
| *E. coli* L-LDH (P33232) |  | 8.947 | 11.376 | 11.316 | 12.698 | 8.724 | 10.649 | 9.948 | 10.158 | 8.269 |
| Pig heart cytoplasmic MDH (P11708) | 8.947 |  | 54.955 | 45.808 | 46.246 | 19.723 | 19.062 | 19.883 | 17.353 | 18.95 |
| *T. thermophilus* MDH (AFH39418.1) | 11.376 | 54.955 |  | 51.829 | 56.402 | 19.54 | 20.958 | 19.048 | 15.821 | 17.804 |
| *L. pneumophila* MDH (Q5ZT13.1) | 11.316 | 45.808 | 51.829 |  | 63.415 | 22.007 | 23.512 | 17.456 | 16.964 | 15.929 |
| *C. burnetii* CBU1241 (NP_820236.1) | 12.698 | 46.246 | 56.402 | 63.415 |  | 21.252 | 23.881 | 18.452 | 18.507 | 18.991 |
| Pig heart mitochondrial MDH (P00346.2) | 8.724 | 19.723 | 19.54 | 22.007 | 21.252 |  | 58.28 | 23.525 | 17.778 | 22.887 |
| *E. coli* MDH (NP_417703.1) | 10.649 | 19.062 | 20.958 | 23.512 | 23.881 | 58.28 |  | 24.242 | 21.713 | 21.622 |
| *B. melitensis* LDH/MDH (3GVH_B) | 9.948 | 19.883 | 19.048 | 17.456 | 18.452 | 23.525 | 24.242 |  | 29.102 | 29.755 |
| Cow heart LDH (AAI42007.1) | 10.158 | 17.353 | 15.821 | 16.964 | 18.507 | 17.778 | 21.713 | 29.102 |  | 35.404 |
| *L. casei* LDH (2ZQY_A) | 8.269 | 18.95 | 17.804 | 15.929 | 18.991 | 22.887 | 21.622 | 29.755 | 35.404 |  |

**(B)**

|  | *L. pneumophila* NAD-ME (Q5ZXT0) | Pigeon liver NADP-ME (P40927.1) | *Lactobacillus casei* MLE (CAQ65929.1) | *Oenococcus oeni* MLE (Q48796.1) | *L. pneumophila* NAD-ME (Q5ZW08) | *C. burnetii* CBU0823  (NP_ 819843.1) | *L. pneumophila* NAD-ME (Q5ZRB1) | *E. coli* NAD-ME (NP_415996.2) |
| --- | --- | --- | --- | --- | --- | --- | --- | --- |
| *L. pneumophila* NAD-ME (Q5ZXT0) |  | 19.323 | 18.898 | 18.701 | 18.982 | 18.591 | 17.808 | 17.255 |
| Pigeon liver NADP-ME (P40927.1) | 19.323 |  | 38.104 | 35.714 | 35.484 | 34.838 | 40.433 | 41.441 |
| *L.casei* MLE (CAQ65929.1) | 18.898 | 38.104 |  | 69.686 | 41.398 | 40.58 | 39.602 | 38.198 |
| *O. oeni* MLE (Q48796.1) | 18.701 | 35.714 | 69.686 |  | 41.865 | 41.651 | 38.574 | 37.179 |
| *L. pneumophila* NAD-ME (Q5ZW08) | 18.982 | 35.484 | 41.398 | 41.865 |  | 54.912 | 46.223 | 44.523 |
| *C. burnetii* CBU0823 (NP_819843.1) | 18.591 | 34.838 | 40.58 | 41.651 | 54.912 |  | 43.525 | 44.326 |
| *L. pneumophila* NAD-ME (Q5ZRB1) | 17.808 | 40.433 | 39.602 | 38.574 | 46.223 | 43.525 |  | 51.619 |
| *E. coli* NAD-ME (NP_415996.2) | 17.255 | 41.441 | 38.198 | 37.179 | 44.523 | 44.326 | 51.619 |  |
